# Delivering Effective Hepatitis C Virus Treatment in an Embedded Primary Care Setting Within a Tertiary Care Hospital in Karachi, Pakistan

**DOI:** 10.1111/jvh.70164

**Published:** 2026-03-12

**Authors:** Sabeen Shah, Nyashadzaishe Mafirakureva, Adam Trickey, Aliya Hasnain, Uzma Khan, Saira Khowaja, Hiba Ashraf, Naila Baig‐Ansari, Matthew Hickman, Peter Vickerman, Josephine G. Walker, Aaron G. Lim

**Affiliations:** ^1^ Global Health Directorate Indus Hospital & Health Network Karachi Pakistan; ^2^ Department of Family Medicine Aga Khan University Karachi Pakistan; ^3^ Population Health Sciences, Bristol Medical School University of Bristol Bristol UK; ^4^ Health Economics and Decision Science, School of Health & Related Research University of Sheffield Sheffield UK; ^5^ Department of Medicine Aga Khan University Karachi Pakistan; ^6^ Interactive Research and Development (IRD) Global Singapore Singapore; ^7^ Department of Epidemiology, Biostatistics and Occupational Health McGill University Montréal Canada; ^8^ TBPeople Vancouver Canada; ^9^ Indus Hospital Research Centre Indus Hospital & Health Network Karachi Pakistan

**Keywords:** direct‐acting antiviral treatment, hepatitis C virus, loss to follow‐up, low middle income countries

## Abstract

Hepatitis C virus (HCV) endemic regions require accessible treatment interventions. Effectiveness and costs of a pilot HCV treatment programme were evaluated at an embedded primary care service within a tertiary care centre at Indus Hospital and Health Network in Karachi, Pakistan. Data on patients (*n* = 1288, median age 40 years) initiating direct‐acting antiviral (DAA) treatment (October 2016 to December 2018) were extracted from hospital records. Eligible patients had chronic HCV, were treatment naïve, and without hepatic decompensation. Multivariable logistic regression analysed factors associated with treatment outcomes (not completing treatment, treatment completion without sustained virological response test at 12 weeks (SVR12) visit, and treatment completion with SVR12). Costs (2019 USD) were estimated using micro‐costing from financial records and staff interviews. Among 1288 patients (63% women), 93% (1200/1288) completed treatment, and 74% (884/1200) attended SVR12 visit, with 98% (*n* = 870/884) cured. Compared with 0–29 year‐olds, incomplete treatment was lower among 30–49 year‐olds (aOR 0.47 [0.26–0.83]) and ≥ 50 year‐olds (aOR 0.48 [0.24–0.93]). SVR12 non‐attendance was higher for 24‐week versus 12‐week regimens (aOR: 3.46 [1.51–7.93]), but lower for patients with APRI scores 0.5–1.49 (aOR 0.69 [0.50–0.96]) and ≥ 1.5 (aOR 0.44 [0.24–0.78]) compared to 0–0.49. The mean treatment cost was $370.74 per patient, driven by clinic visits $271.80 (73.3%), labs $68.32 (18.4%), and DAAs $30.62 (8.3%). Overall, a high treatment completion and cure rate were achieved, with a low average cost per patient, indicating that this HCV treatment model can be affordable and may be considered for widescale treatment scale‐up in Pakistan.

**Abbreviations**
APRI: Aspartate Aminotransferase to Platelet Ratio Index
CLIA: chemiluminescence immunoassay
CTP: *Child*‐*Turcotte‐*Pugh
DAA: direct acting antiviral
DALY: disability‐adjusted life‐year
ETR: End of Treatment Response
HbA1c: Haemoglobin A1c
HCV: hepatitis C virus
HIV: human immunodeficiency virus
HMIS: Hospital Management Information System
IHHN: Indus Hospital and Health Network
LMIC: low middle‐income country
LTFU: lost to follow up
MSF: Médecins Sans Frontières
PCR: polymerase chain reaction
QALY: quality‐adjusted life year
SVR 12: sustained virological response 12 weeks post‐treatment completion
TSH: thyroid stimulating hormone
USD: US dollars
WHO: World Health Organisation

## 1 Introduction

Pakistan's expanding hepatitis C virus (HCV) epidemic makes up over 10% of the global HCV disease burden, with an estimated 7.3 million people having chronic infection in 2018 [1, 2, 3]. Increasing accessibility to highly effective curative HCV treatment presents an opportunity for healthcare providers in Pakistan to reduce HCV‐related morbidity and mortality, which is expected to rise if treatment is not scaled‐up [2, 4, 5, 6]. The existing HCV treatment programmes across the country are limited by inadequate health infrastructure and barriers in accessing resources for HCV diagnosis and treatment [7, 8].

As Pakistan strives towards achieving the World Health Organization (WHO) targets for HCV elimination as a public health problem by 2030 [7], it is crucial for all healthcare providers within Pakistan to contribute to the effort. To complement national guidance [9], showcasing real‐world examples of good practice and transparency on the resource requirements and effectiveness of implementing HCV treatment services can offer useful perspectives. Insights gained from existing programmes can provide supporting evidence to local healthcare providers on how best to integrate HCV treatment services into existing practices.

Indus Hospital and Health Network (IHHN) is a growing network of hospitals and clinics spread across Pakistan providing high‐quality health services free‐of‐charge to millions of patients, focusing on the most in‐need patient populations. To address the high HCV burden and lack of treatment availability, IHHN initiated a pilot HCV Control Programme in October 2016. The service was integrated into a primary care clinic embedded in a tertiary care centre in Korangi, a low socio‐economic area in Karachi, Pakistan. This programme was geared towards providing laboratory tests, doctor consultations, and treatment services. This pilot operated until December 2018, after which the programme was expanded to primary care clinics across urban and rural sites across the Sindh and Punjab provinces [10].

This paper evaluates the cascade of care and costs of delivering a HCV treatment intervention as part of the pilot HCV Control Programme at IHHN. Cascade stages considered include (1) baseline medical evaluation of HCV polymerase chain reaction (PCR) test positive patients, (2) initiation on treatment, (3) completing treatment, and (4) achieving sustained virological response 12 weeks post treatment completion (SVR12).

## 2 Methods

### 2.1 Study Design and Setting

This was a retrospective cohort study among patients with chronic HCV enrolled in the pilot HCV Control Programme from October 2016 to December 2018 at the Indus Hospital, Korangi campus in Karachi, Pakistan. HCV screening at the family medicine clinic at Indus Hospital was either a symptom‐based, non‐systematic process dependent on the clinical judgement of family medicine doctors or was done based on previous diagnosis. Screening involved the use of a laboratory‐based chemiluminescence immunoassay (CLIA) test. Patients who tested positive for HCV antibodies at screening were offered diagnostic testing using PCR tests during their subsequent family medicine clinic visit. Patients with confirmed HCV infection were referred to the HCV clinic for a focused medical evaluation from a dedicated clinical team consisting of one nurse and a doctor. Both team members were trained on programmatic clinical protocol, data collection tools and patient education materials.

Treatment start dates were divided into two periods to coincide with changes in HCV treatment protocol, based on the direct acting antiviral (DAA) regimen prescribed. Between October 2016 and November 2017 adults (age ≥ 18 years), who were treatment naïve, HCV PCR positive, *Child*‐*Turcotte*‐Pugh (CTP) class A patients with genotype 1,2 or 3, were eligible for treatment initiation through the HCV clinic. These patients were assessed for liver disease progression using Aspartate Aminotransferase to Platelet Ratio Index (APRI) score.

An individual's HCV genotype result guided treatment duration and combination of medications prescribed based on WHO guidelines [11]. Genotype 3 patients were prescribed sofosbuvir (400 mg/day) and weight‐based ribavirin (1000 to 1200 mg/day) for 24 weeks. Genotype 1 patients were prescribed sofosbuvir (400 mg/day), weight‐based ribavirin (1000 to 1200 mg/day) and pegylated interferon (180 μg/week) for 12 weeks. Genotype 2 patients were prescribed sofosbuvir 400 mg and weight‐based ribavirin (1000 to 1200 mg/day) for 12 weeks.

**FIGURE 1:**
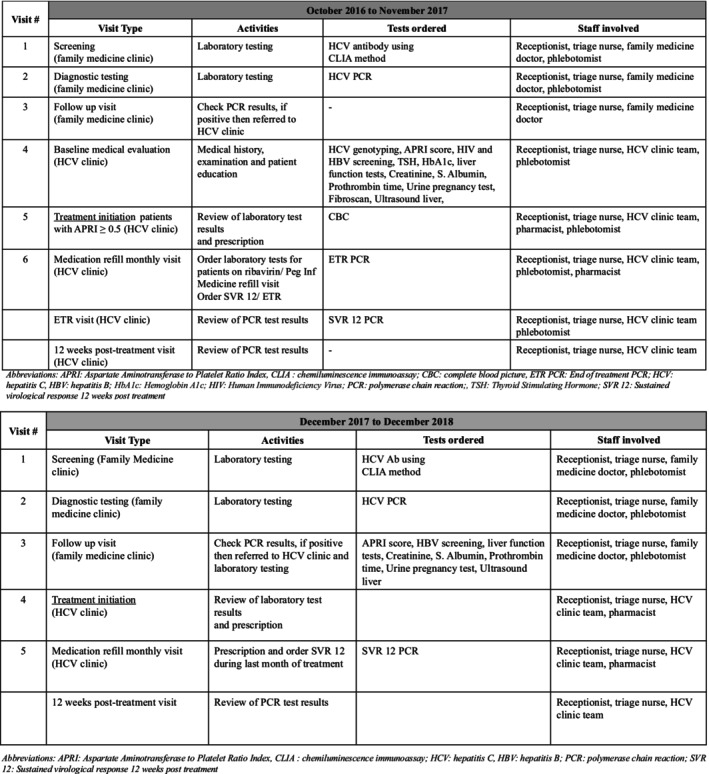
(a) Schematic treatment pathway October 2016 to November 2017. (b) Schematic treatment pathway December 2017 to December 2018.

Between December 2017 and December 2018 there were five major changes in clinical protocol (Figure 1a,b). These were: (1) reducing the list of laboratory tests performed based on their limited value in guiding subsequent management decisions; HCV genotyping, human immunodeficiency virus (HIV) screening, thyroid stimulating hormone (TSH), Haemoglobin A1c (HbA1c), Fibroscan and End of Treatment Response (ETR) PCR, were discontinued; (2) treatment was offered to all patients irrespective of APRI score including those with past history of treatment; (3) treatment regimen shifted to 12 weeks of sofosbuvir (400 mg/day) and daclatasvir (60 mg/day) combination therapy for all patients except those with compensated cirrhosis for whom weight based ribavirin (1000 to 1200 mg/day) was added and treatment total duration extended to 24 weeks (patients with decompensated cirrhosis were managed in gastroenterology clinic); (4) reduction in number of pre‐treatment initiation visits from three to two visits for HCV antibody positive patients; whilst the number of on‐treatment visits remained unchanged and dependent on total duration of treatment; and (5) all family medicine nurses and doctors were trained to provide algorithm‐based care.

Due to limited medication availability, treatment initiation was prioritised for patients with APRI ≥ 0.5 (Metavir stage > F2), while patients with APRI < 0.5 were deferred for treatment and kept on regular six‐monthly monitoring to assess liver cirrhosis staging through APRI score. In addition to medication prescription, the clinical team focussed on educating patients about the importance of compliance to treatment, regular clinic visits for medication refill and medical evaluation, and use of long‐term contraception for patients prescribed ribavirin‐based regimens (Figure 1). Detailed referral criteria for inclusion in the treatment programme and changes in HCV treatment protocol during the two time periods can be found in the Supporting Information.

### 2.2 Data Sources

Patient‐level data relevant to patients' involvement in the HCV Control Programme were extracted from the electronic Hospital Management Information System (HMIS) database, which contains health information on every patient registered at IHHN. Data were captured on treatment regimen and duration, as well as APRI score, age, and sex. Information on previous treatment, comorbidities, previous surgery, addictions, and schooling was self‐reported by the patient. All patient‐level data covered the observation period plus 9 months afterwards (i.e., from October 2016 to September 2019) to capture follow‐up patient outcomes and resource use. Patient‐level data was de‐identified prior to any analysis to ensure patient confidentiality and anonymity.

### 2.3 Statistical Analysis

The endpoints considered were (1) incomplete treatment, (2) completed treatment but did not attend the SVR12 visit, and (3) completed treatment and had PCR test for SVR12 response. Differences in outcomes (not completing treatment, treatment completion without SVR12 visit, and treatment completion with SVR12) by patient and treatment characteristics were analysed using Chi‐square tests. These patient and treatment characteristics were time of starting treatment, regimen prescribed, treatment duration, APRI score, age, sex, schooling, previous HCV treatment history, comorbidity status, previous history of surgery, and any substance addiction. Associations between these characteristics and not completing treatment were analysed using logistic regression. Similarly, among those that completed treatment, patient and treatment characteristics associated with not attending SVR12 appointment were analysed using logistic regression. The logistic regression analyses were not performed separately for the two treatment start periods due to a lack of power in the first period.

### 2.4 Costing Analysis

The cost analyses followed an ingredients‐based approach, involving the identification, quantification, and valuation of all resources (direct medical, indirect and overheads) used in the treatment and follow‐up for each patient in the cohort (Supporting Information). Resources used (including facility visits, medicines, and laboratory tests) were obtained from the HMIS database. Unit costs were applied to patient‐level resource use (e.g., in terms of type and frequency of visit or test) to obtain the total cost for each patient (see Supporting Information for further details). Unit costs were estimated using cost data obtained from a detailed review of IHHN's financial records and supplemented by interviews with key technical staff involved in the planning, implementation, and coordination of the programme. All costs were gathered in the local currency (Pakistani Rupee), adjusted for inflation to 2019 prices using the Consumer Price Indices for Pakistan and then converted to US dollars (USD) using the 2019 average market‐based exchange rate (1 USD = 136 Pakistani Rupee) [12, 13]. A healthcare provider's perspective was used in estimating costs.

## 3 Results

### 3.1 Patient Characteristics

Table 1 presents the characteristics of the 1288 patients in the HCV cohort that started treatment. Excluding 101 patients with missing APRI score, the median recorded APRI score was 0.47 (interquartile range [IQR]: 0.27–0.90), whilst the median age was 40 years (IQR: 32–50). Most patients were females (*n* = 814, 63.2%) and were recorded as having ever been to school (*n* = 684, 53.1%). There were 123 (9.6%) patients who were recorded as previously having had HCV treatment. In total, 306 (23.8%) patients had a comorbidity reported, whilst 532 (41.3%) had a previous surgery recorded, and 258 (20.0%) reported having a substance use addiction.

**TABLE 1:** Patient characteristics.

Characteristic	Overall	Did not complete treatment	Completed treatment *N* = 1200 (93.2%)		*χ* ^2^ test for differences, *p*
			Did not attend SVR12	Attended SVR12	
	*N* = 1288 (100%)	*N* = 88 (6.8%)	*N* = 316 (24.5%)	*N* = 884 (68.6%)	
Treatment start period					
October 2016–November 2017	229 (17.8%)	9 (3.9%)	11 (4.8%)	209 (91.3%)	< 0.001
December 2017–December 2018	1059 (82.2%)	79 (7.5%)	305 (28.8%)	675 (63.7%)	
Regimen prescribed					
SOF/DAC	889 (69.0%)	54 (6.1%)	236 (26.6%)	599 (67.4%)	< 0.001
SOF/RBV	212 (16.5%)	8 (3.8%)	13 (6.1%)	191 (90.1%)	
SOF/DAC/RBV	178 (13.8%)	24 (13.5%)	66 (37.1%)	88 (49.4%)	
SOF/RBV/Peg‐INF	9 (0.7%)	2 (22.2%)	1 (11.1%)	6 (66.7%)	
Treatment duration					
12 weeks	891 (69.2%)	53 (6.0%)	233 (26.2%)	605 (67.9%)	0.037
24 weeks	397 (30.8%)	35 (8.8%)	83 (20.9%)	279 (70.3%)	
APRI score					
0–0.49	612 (47.5%)	38 (6.2%)	164 (26.8%)	410 (67.0%)	0.134
0.5–1.49	438 (34.0%)	26 (5.9%)	97 (22.2%)	315 (71.9%)	
≥ 1.5	137 (10.6%)	16 (11.7%)	29 (21.2%)	92 (67.2%)	
Missing	101 (7.8%)	8 (7.9%)	26 (25.7%)	67 (66.3%)	
Age (years)					
0–29	236 (18.3%)	22 (9.3%)	50 (21.2%)	164 (64.5%)	0.194
30–49	712 (55.3%)	41 (5.8%)	188 (26.4%)	483 (67.8%)	
≥ 50	340 (26.4%)	25 (7.4%)	78 (22.9%)	237 (69.7%)	
Sex					
Male	474 (36.8%)	29 (6.1%)	132 (27.9%)	313 (66.0%)	0.098
Female	814 (63.2%)	59 (7.3%)	184 (22.6%)	571 (70.2%)	
Schooling					
None	487 (37.8%)	35 (7.2%)	119 (24.4%)	333 (68.4%)	0.003
Been to school	684 (53.1%)	42 (6.1%)	153 (22.4%)	489 (71.5%)	
Missing	117 (9.1%)	11 (9.4%)	44 (27.6%)	62 (53.0%)	
Previous HCV treatment					
No	1039 (80.7%)	66 (6.4%)	241 (23.2%)	732 (70.5%)	0.013
Yes	123 (9.6%)	12 (9.8%)	30 (24.4%)	81 (65.9%)	
Missing	126 (9.8%)	10 (7.9%)	45 (35.7%)	71 (56.4%)	
Any comorbidity					
No	835 (64.8%)	49 (5.9%)	191 (22.9%)	595 (71.3%)	0.009
Yes	306 (23.8%)	29 (9.5%)	75 (24.5%)	202 (66.0%)	
Missing	147 (11.4%)	10 (6.8%)	50 (34.0%)	87 (59.2%)	
Previous surgery					
No	586 (45.5%)	43 (7.3%)	151 (25.8%)	392 (66.9%)	0.001
Yes	532 (41.3%)	34 (6.4%)	105 (19.7%)	393 (73.9%)	
Missing	170 (13.2%)	11 (6.5%)	60 (35.3%)	99 (58.2%)	
Any addiction					
No	868 (67.4%)	58 (6.7%)	202 (23.3%)	608 (70.1%)	0.033
Yes	258 (20.0%)	19 (7.4%)	58 (22.5%)	181 (70.2%)	
Missing	162 (12.6%)	11 (6.8%)	56 (34.6%)	95 (58.6%)	

Abbreviations: APRI, Aspartate Aminotransferase to Platelet Ratio Index; DAC, daclatasvir; LTFU, loss to follow‐up; Peg‐INF, pegylated interferon; RBV, ribavirin; SOF, sofosbuvir; SVR, sustained virological response.

### 3.2 Cascade of Care

Overall, 229 (17.8%) patients started in the first treatment period and 1059 (82.2%) started treatment in the second treatment period when sofosbuvir‐daclatasvir became available for prescription through IHHN from December 2017. The majority (*n* = 889; 69.0%) of patients were prescribed sofosbuvir with daclatasvir, whilst 212 (16.5%) were prescribed sofosbuvir with ribavirin, 178 (13.8%) were prescribed sofosbuvir with daclatasvir and ribavirin, and only 9 (0.7%) of patients were prescribed sofosbuvir, ribavirin, and peg‐interferon (Table 1). Of all 1288 patients, 891 (69.2%) and 397 (30.8%) were on a 12‐week and 24‐week regimen, respectively.

**FIGURE 2:**
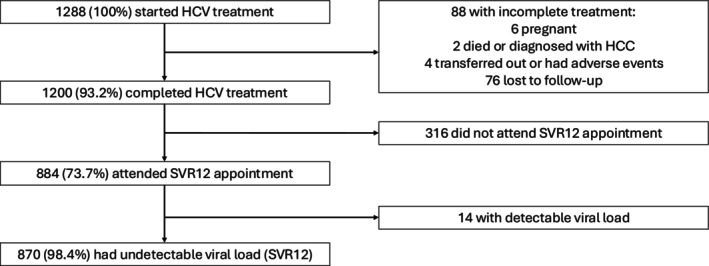
Flow chart of patient outcomes. HCC, hepatocellular carcinoma; HCV, hepatitis C; LTFU, loss to follow‐up; SVR 12, sustained virological response 12 weeks post treatment.

The treatment cascade of care for the 1288 patients that started HCV treatment is summarised in Figure 2. Of the 1288 that started treatment, 88 (6.8%) did not complete their treatment. Of these 88, 6 (6.8%) became pregnant during treatment, 2 (2.3%) either died or were diagnosed with hepatocellular carcinoma, 4 (4.6%) stopped due to transfer out or adverse events, and 76 (86.4%) were lost to follow up (LTFU). Of the 1200 (93.2%) that completed treatment, 316 (26.3%) did not attend their SVR12 follow‐up appointment. Of the 884 (73.7%) that attended their SVR12 follow‐up appointment, 870 (98.4%) had an undetectable viral load.

Table 1 also shows the differences in the proportions of patients achieving different outcomes by their characteristics, as well as treatment start period, treatment duration, and regimen prescribed. The biggest differences in outcomes were seen based on the time of starting treatment and the regimen prescribed. A higher percentage (91.3%) of those who started treatment between October 2016 and November 2017 had completed treatment and had an SVR12 appointment than those who started between December 2017 and December 2018 (63.7%). Similarly, 90.1% of people that were treated with sofosbuvir‐ribavirin had completed treatment and had an SVR12 appointment than those starting on other regimens (< 67.5%). This correlates with the treatments available in each treatment start period.

### 3.3 Predictors of Incomplete Treatment

In multivariable analyses (Table 2), there was no evidence of differing odds of not completing treatment for people starting on SOF/DAC, SOF/RBV, or SOF/DAC/RBV. However, there was some evidence that people who were treated with SOF/RBV/Peg‐INF had higher odds of not completing treatment than those prescribed SOF/DAC (adjusted odds ratio [aOR]: 10.01, 95% confidence interval [CI]: 0.98–102.24). Age was associated with incomplete treatment, with patients aged 30–49 years (aOR: 0.47, 95% CI: 0.26–0.83) and ≥ 50 years (aOR: 0.48, 95% CI: 0.24–0.93) being more likely to complete treatment than those aged 0–29 years. In addition, there was also some evidence that patients with a comorbidity were less likely to complete treatment than those without a comorbidity (aOR: 1.63, 95% CI: 0.98–2.70). There was no evidence that treatment duration, APRI score, sex, history of schooling, previous HCV treatment, previous surgery, or substance use addiction were associated with not completing treatment.

**TABLE 2:** Adjusted odds ratios for (a) not completing treatment among the *N* = 1288 that started treatment and (b) not attending an SVR12 appointment among the *N* = 1200 that completed treatment.

	(a) Not completing treatment		(b) Completing treatment and not attending an SVR12 appointment	
	Adjusted odds ratio (95% CI)	*p*	Adjusted odds ratio (95% CI)	*p*
Treatment start period				
October 2016–November 2017	1		1	
December 2017–December 2018	5.84 (1.07–31.92)	0.042	23.23 (5.97–90.46)	< 0.001
Regimen prescribed				
SOF/DAC	1		1	
SOF/RBV	1.14 (0.23–5.64)	0.871	0.93 (0.31–2.80)	0.894
SOF/DAC/RBV	0.98 (0.32–2.98)	0.970	1.00 (0.42–2.35)	0.998
SOF/RBV/Peg‐INF	10.01 (0.98–102.24)	0.052	3.41 (0.29–40.21)	0.330
Treatment duration				
12 weeks	1		1	
24 weeks	2.47 (0.84–7.25)	0.101	3.46 (1.51–7.93)	0.003
APRI score				
0–0.49	1		1	
0.5–1.49	0.94 (0.54–1.63)	0.809	0.69 (0.50–0.96)	0.029
≥ 1.5	1.36 (0.64–2.92)	0.426	0.44 (0.24–0.78)	0.005
Missing	1.23 (0.54–2.81)	0.628	0.66 (0.39–1.11)	0.118
Age (years)				
0–29	1		1	
30–49	0.47 (0.26–0.83)	0.009	1.21 (0.82–1.79)	0.330
≥ 50	0.48 (0.24–0.93)	0.030	0.87 (0.55–1.36)	0.532
Sex				
Male	1		1	
Female	1.33 (0.78–2.25)	0.290	0.77 (0.56–1.05)	0.097
Schooling				
None	1		1	
Been to school	0.92 (0.55–1.52)	0.735	0.86 (0.63–1.17)	0.332
Missing	6.71 (0.89–50.86)	0.066	2.22 (0.62–7.95)	0.220
Previous HCV treatment				
No	1		1	
Yes	1.22 (0.98–2.70)	0.584	0.72 (0.44–1.18)	0.193
Missing	2.10 (0.03–147.50)	0.732	0.62 (0.13–2.97)	0.550
Any comorbidity				
No	1		1	
Yes	1.63 (0.98–2.70)	0.058	1.16 (0.83–1.62)	0.390
Missing	0.11 (0.00–10.67)	0.342	0.55 (0.14–2.28)	0.413
Previous surgery				
No	1		1	
Yes	0.78 (0.47–1.30)	0.343	0.74 (0.54–1.01)	0.057
Missing	NA	NA	2.50 (0.56–11.14)	0.231
Substance use addiction				
No	1		1	
Yes	1.47 (0.81–2.66)	0.203	1.21 (0.83–1.79)	0.322
Missing	NA	NA	0.70 (0.12–4.21)	0.694

Abbreviations: APRI, Aspartate Aminotransferase to Platelet Ratio Index; CI, confidence interval; DAC, daclatasvir; Peg‐INF, pegylated interferon; RBV, ribavirin; SOF, sofosbuvir; SVR, sustained virological response.

### 3.4 Predictors of Not Attending Post‐Treatment SVR12 Visit

In multivariable analyses (Table 2), among patients who completed treatment, those starting treatment in the later start period had higher odds of not attending their SVR12 appointment than those starting in the earlier period (aOR: 23.23, 95% CI: 5.97–90.46). There was no evidence of differences in odds of not attending an SVR12 appointment by regimen. However, patients on 24‐week treatment schedules had higher odds of not attending SVR12 appointments (aOR: 3.46, 95% CI: 1.51–7.93). Patients with APRI scores 0.5–1.49 (aOR: 0.69, 95% CI: 0.50–0.96) and ≥ 1.5 (aOR: 0.44, 95% CI: 0.24–0.78) were less likely to be LTFU before their SVR12 appointment than those with APRI scores 0–0.49. There was evidence in univariable analyses that females were less likely to miss their SVR12 appointment than males (Table S4); however, this association attenuated in multivariable analyses. In both univariable and multivariable analyses, there was some evidence that patients who had previous surgery were less likely to miss their SVR12 appointment than those that had not previously had surgery (aOR: 0.74, 95% CI: 0.54–1.01). There was no evidence of multivariable associations between post‐treatment LTFU and age, history of schooling, previous HCV treatment, having a comorbidity, or having any substance use addiction.

### 3.5 Cost of HCV Treatment

The mean cost per patient treated for HCV was estimated to be $370.74 (SD = $27.21), with the total cost of HCV treatment for the 1288 people who initiated treatment being $477,513.12. Therefore, the cost per person completing treatment (*n* = 1200) was estimated to be $397.93.

**FIGURE 3:**
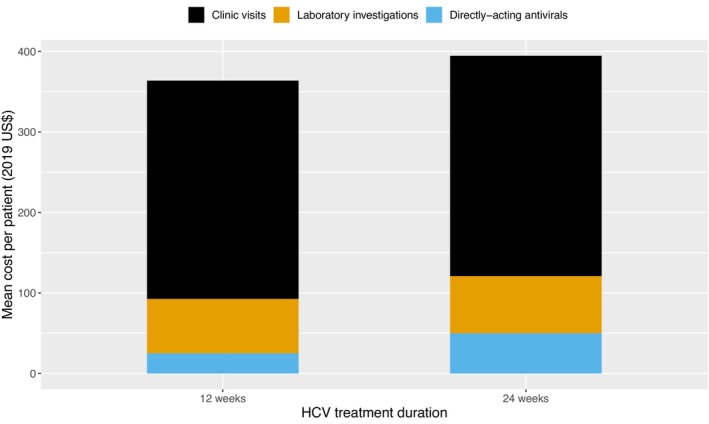
Mean HCV treatment costs per patient by treatment duration.

The major cost driver was the cost of clinic visits, $271.80 (SD = $2.85), contributing 73.3% of the total HCV treatment cost (Table 3). This clinic visit cost was primarily due to medical consultations (62.7%), support personnel (17.1%), and coordination (16.9%) costs, combined with low patient numbers making up clinic overhead costs (Tables S6–S8). The other cost drivers were laboratory investigations costs ($68.32, SD = $23.20) and DAA drug costs ($30.62, SD = $10.44), contributing 18.4% and 8.3% of the total HCV treatment costs, respectively. The distribution of mean costs per patient for different APRI scores and cost drivers is shown in Table 3. The total HCV treatment costs varied by treatment duration, with higher costs for 24 weeks ($394.72, SD = $24.46) compared to 12 weeks ($363.75, SD = $23.79) of DAA treatment (Figure 3). Treatment costs were slightly higher (by approximately $10–20) for patients with an APRI score larger than 0.49, compared to those with a lower APRI score below 0.49 (Figure S2), largely due to differences in the costs of DAA medicines as those with larger APRI scores are more likely to be prescribed DAA treatment for 24 weeks instead of 12 weeks.

**TABLE 3:** Mean per‐patient cost of HCV treatment using DAA‐based regimens. Costs are presented as mean (standard deviation) in 2019 United States dollars.

Cost category	APRI score				
	0–0.49	0.5–1.49	≥ 1.5	Missing	Full cohort
Clinic visits	271.6 (2.58)	272.18 (2.75)	272.19 (4.01)	271.12 (2.9)	271.8 (2.85)
Laboratory investigations	66.01 (23.3)	70.07 (22.36)	70.5 (20.69)	71.56 (27.03)	68.32 (23.2)
DAA medicines	27.62 (7.67)	32.34 (11.41)	41.72 (11.91)	28.41 (8.6)	30.62 (10.44)
Total	365.23 (27.08)	374.59 (25.93)	384.41 (20.07)	371.09 (31.44)	370.74 (27.21)

Abbreviations: APRI: Aspartate Aminotransferase to Platelet Ratio Index; DAA: directly acting antivirals; HCV: hepatitis C virus.

## 4 Discussion

In this study, we conducted a secondary analysis of programmatic and costing data derived from a pilot HCV Control Programme in Pakistan, covering the period from October 2016 to December 2018. The service was integrated into a primary care clinic embedded within a tertiary care centre in Korangi, and HCV treatment was delivered by a private health care provider in a low‐ and middle‐income country (LMIC) where poor health systems and socio‐economic factors impede access to care. Our results demonstrated a high treatment completion rate (93.2%) among those who started treatment, and a high cure rate (98.4%) among those that attended their SVR12 appointment. We estimated total treatment costs for the full cohort to be US $370.74 per patient, with clinic visit costs being the main driver for the cost of care $271.80 (73.3%), followed by laboratory investigations $68.32 (18.4%) and DAA medicines $30.62 (8.3%). Overall, our analyses suggest that HCV treatment services in this urban, general population, hospital‐based primary care setting in Pakistan can be a highly effective and low‐cost intervention which is similar to treating one patient living with the chronic condition type 2 diabetes mellitus each year (reported as being $235 USD annually in 2023) [14]. Moreover, our results suggest that treating HCV would be equivalent to approximately 3 months of the national minimum wage (PKR 17,500 per month in 2019, or ~$130 USD) for a one‐time curable illness, unlike chronic diseases that require ongoing expenditure. Considering that the major cost was clinic visits, which is higher in a tertiary care facility versus primary care facility, this can potentially improve the cost per patient treated or cured when delivered in community‐based primary care clinics. Our findings may be generalisable to other settings with similar capacity for HCV treatment delivery.

We found that patients starting treatment in the later period were less likely to have completed treatment or attend their SVR12 appointment, likely because they had less time to progress through the HCV cascade of care. Additionally, we found that people on peg‐interferon regimens had lower odds of completing treatment, and those on longer treatment schedules were more likely to miss their SVR12 appointment. Our results also showed that patients with APRI scores above 0.5 and previous surgery were more likely to attend their SVR visit. However, since only clinically stable patients were considered for treatment initiation, the observed treatment‐seeking behaviour of patients with higher APRI scores may not be linked with being unwell. It is likely that the doctor's counselling about disease severity influenced patients' attitudes towards treatment adherence.

Based on the high cure rates found among the individuals that attended their SVR12 appointment, no additional efforts were made to actively track and test those patients that did not attend their SVR12 visit. Given the well‐established efficacy of pangenotypic DAAs [15] and the high SVR rates observed in our study, it was assumed that a substantial proportion of treated patients who did not attend their SVR12 visit also achieved cure. However, as SVR12 testing was not conducted in approximately one‐quarter (26.3%) of treated patients, this assumption remains speculative, and the absence of follow‐up testing in this group represents a limitation of the study.

The reasons for patients completing treatment but not attending their SVR12 visit are multifactorial including provision of facility‐based care with no incentives. Other contributing factors may include time and expenses required to travel to clinic, work commitments especially for daily wagers, and the generally asymptomatic nature of chronic HCV infection prior to decompensated liver cirrhosis, which may lead some patients to believe that completing treatment was sufficient and prompting them to skip the SVR12 visit. Moreover, there were few patient‐level characteristics that could be included in the regression analyses to understand what reduces treatment completion or chance of attending the SVR12 visit, limiting our ability to identify more tailored treatment and testing prioritisation strategies to minimise LTFU in this setting, as adopted by national level programmes in other countries [16]. Nevertheless, in our study we found that few patients (< 8%) did not complete treatment. Meanwhile, considering the high efficacy of pangenotypic DAAs, sofosbuvir and daclatasvir which, in our study, resulted in a high cure rate of 98.4% among those who completed treatment and attended their SVR12 appointment, it is reasonable to assume that a similarly high proportion of those who completed treatment but did not return for confirmatory SVR12 testing would have been cured.

### 4.1 Comparison With Other Literature

To date, few HCV treatment effectiveness studies based on real‐world data have been published from LMICs [2, 17, 18]. A cure rate of 84% has been reported from a predominantly genotype 3 infected population residing in an urban slum of Pakistan [19], whereas another study from Cambodia reported a cure rate of 96.8% in a predominantly genotype 1 infected population [20]. Our results demonstrated a high cure rate (98.4%), which is comparable to rates reported from clinical trials, real‐world clinical practice settings and public health programmes [21, 22, 23], but rates for attending the SVR12 appointments were low (73.7%). A multi‐country cohort analysis from five LMICs [24] also reported high treatment completion and cure rates, consistent with our findings. Although our study was not conducted in public sector settings, the outcomes were similar. Notably, as a private‐sector non‐governmental organisation (NGO) run programme, our results remain encouragingly high. Boeke et al. [24] also reported that 24‐week treatment duration, younger patient age, and later treatment initiation were associated with lower treatment completion, findings that align with our study. In another study from Rwanda [25], a lower proportion of people returned for their SVR12 visits compared with our cohort, though their reported cure rate was higher.

Existing literature from LMICs highlight treatment costs from a HCV clinic in an urban slum of Pakistan operated by the NGO Médecins Sans Frontières (MSF) [18] and a clinic located in the gastroenterology department of a tertiary care hospital of Cambodia [20]. Total per patient treatment costs of our IHHN HCV treatment programme are comparable to those reported by the MSF‐run clinic [18] ($344.16 versus $370.74 at IHHN); however, the MSF‐run clinic reported DAAs contributing 52% to the total cost of treatment, whereas in our study clinic visit costs (73%) were the main driver of costs in the IHHN HCV treatment programme, with only 8.3% of total treatment costs being attributable to the cost of DAAs. Meanwhile, the clinic visit costs reported by the MSF‐run clinic contributed 22% ($159.87) to the overall treatment cost. Large patient numbers attending the MSF‐run clinic are likely the cause for lower per patient costs, suggesting that an increase in patient volume at the IHHN program could similarly result in reduced treatment costs per patient. In the Cambodian tertiary hospital setting [20], the authors reported findings from a full model of care and a simplified model of care for HCV treatment in Cambodia, with the median total cost of HCV testing and treatment being $925 for the full model and $376 for the simplified model. The biggest contributor to the overall cost in both models was DAA costs, making up 26% and 42% of the total treatment cost for the full model and simplified model, respectively [20]. Even though the treatment pathways vary, our model of care closely represents Pakistan's national hepatitis control programme in terms of the general number of appointments and number of laboratory investigations; hence the costs are likely to be similar to the national hepatitis control programme. Conversely, a study from Myanmar [26] reported lower overall treatment costs; however, their estimates excluded overhead expenses, and the costs of individual components such as medications and HCV RNA testing were higher. In our case, the largest cost driver was medical consultations, which inflated clinic visit costs, as patient numbers were limited and physician time remained an expensive resource. Increasing patient enrolment could have the potential to reduce these costs without requiring additional resources.

### 4.2 Strengths and Limitations

This study draws major strength from utilising real‐world implementation data for patient outcomes and costs from a resource‐limited setting. This includes a full costing analysis of a HCV treatment programme implementation. It enabled collection of patient‐level data on resources used to estimate the full costs associated with DAA‐based HCV treatment. Findings from our study allowed us to carefully investigate the complete HCV treatment pathway along with the costs of each component, which can suggest ways that we can improve efficiencies or reduce costs along the pathway. Secondly, our detailed analyses share findings valuable for programme implementers such as younger age being associated with a higher likelihood of incomplete treatment. These insights are invaluable for programme planners to strategise focussed interventions for improved treatment retention rates.

Our study contains several potential limitations. Our costing was based on a cost‐per‐patient treated, but not in terms of cost per quality‐adjusted life year (QALY) gained or cost per disability‐adjusted life‐year (DALY) averted, making it difficult to compare our findings with other settings—although the overall costs per cure were low compared to interventions conducted in other LMICs and similar to other analyses in Pakistan [18, 20].

Our analysis does not include the costs of screening and linkage to treatment, and neither does it consider additional direct non‐medical costs borne by patients (such as travel costs), meaning that it underestimates the full treatment initiation cost [27, 28]. However, our full cost analysis based on ‘real‐world’ data has allowed us to characterise the main healthcare system costs and major cost drivers. These costs are likely to be more relevant to planning and budgeting for initiating or scaling up similar interventions across the country.

This programme was implemented within an NGO‐run primary care service embedded in a tertiary care hospital network providing hepatitis C services free of charge. While the model was designed to align with Pakistan's national hepatitis C elimination strategy [29], the specific environment and observed treatment outcomes and cost estimates may not be directly replicable in other settings such as under‐resourced public‐sector facilities or rural primary care settings, where constraints related to staffing, diagnostics, patient follow‐up, and health system capacity are more pronounced. Replication in such settings would likely require context‐specific adaptations, and further implementation research is needed to evaluate feasibility, effectiveness, and costs in lower‐resource environments.

Moreover, because our HCV treatment intervention was a primary care service integrated into a hospital setting, this required existing infrastructure. Our analysis is unable to extrapolate economic benefits of further integration; however, recent modelling studies have suggested that integration can lead to reductions in overall costs [30].

Finally, we hypothesise that an increase in patient volume may reduce the per‐patient cost of the IHHN pilot programme; however, uncertainties regarding the scaling of other cost components prevent us from accurately estimating the hypothetical cost of treatment per patient under significantly higher enrolment scenarios.

## 5 Conclusion

In this pilot HCV control programme, we presented a disease control model that works closely with primary care providers in a LMIC setting. This successful pilot project demonstrated promising results with desirable treatment outcomes in terms of high treatment completion rates and high cure rates through a low‐cost model. The high proportion of costs from overheads related to clinic visits could have been mitigated through increase in enrolment rate or integrating the programme with other public health programmes. Achieving this would involve strategies like bulk procurement to reduce medication costs and obtaining stakeholder buy‐in to support the process of integration. Despite this, HCV treatment through this programme represents a one‐time investment of about 3 months' national minimum wage that cures the disease, unlike chronic conditions such as diabetes mellitus, which require ongoing annual expenditures. Hence, treatment of HCV could be considered for widescale treatment scale‐up in Pakistan.

## Funding

This work was supported by the University of Bristol's Quality Related (QR) Global Challenges Research Fund (GCRF) strategy funded by Research England.

## Ethics Statement

Permission to carry out the study and conduct analyses on the retrospective cohort data was approved by the Institutional Review Board (IRB) at Interactive Research and Development (IRD) Global (#IRD_IRB_2019_01_004).

## Consent

Given the retrospective nature of the study, the requirement for informed consent for patient data was waived by the ethics committee. All patient data were anonymized and de‐identified prior to analysis to ensure confidentiality and privacy. However, informed consent was obtained while interviewing the staff.

## Conflicts of Interest

J.G.W. and P.V. have received research funding from Gilead Sciences unrelated to this work. The other authors declare no conflicts of interest.

## Supporting information


**Figure S1:** Estimated timing of activities.
**Figure S2:** Mean HCV treatment unit costs by APRI scores.
**Figure S3:** Mean HCV treatment unit costs by METAVIR stages.
**Figure S4:** Mean HCV treatment unit costs by treatment duration or reason for stopping treatment.
**Table S1a:** HCV clinic visit details—treatment time period Oct 2016 to Nov 2017—Non‐Genotype 3 patients.
**Table S1b:** HCV clinic visit details—treatment time period Oct 2016 to Nov 2017—Genotype 3 patients.
**Table S2:** HCV clinic visit details—treatment time period Dec 2017 to Dec 2018.
**Table S3:** Odds ratios for not completing treatment.
**Table S4:** Odds ratios for not attending an SVR12 appointment.
**Table S5:** Staff activities.
**Table S6:** Activities, resources and estimated unit costs.
**Table S7:** Estimated unit costs for clinic visits, laboratory tests and medicines.
**Table S8:** Breakdown for fixed costs for HCV clinic overheads.
**Table S9:** Mean per‐patient cost for clinic visits overall and by APRI score.
**Table S10:** Resource use—HCV laboratory investigations.

## Data Availability

Some of the datasets generated and/or analysed during the current study have been included as summary data in the Supporting Information. Some of the financial records are sensitive and are available upon reasonable request.
