# Supplementary material for: Delivering Effective Hepatitis C Virus Treatment in an Embedded Primary Care Setting Within a Tertiary Care Hospital in Karachi, Pakistan
Source: J Viral Hepat. 2026 Mar 12;33(4):e70164. doi: 10.1111/jvh.70164 (PMC12980558; doi:10.1111/jvh.70164)
Supplement: Supplementary file 1 — Figure S1: Estimated timing of activities. Figure S2: Mean HCV treatment unit costs by APRI scores. Figure S3: Mean HCV treatment unit costs by METAVIR stages. Figure S4: Mean HCV treatment unit costs by treatment duration or reason for stopping treatment. Table S1a: HCV clinic visit details—treatment time period Oct 2016 to Nov 2017—Non‐Genotype 3 patients. Table S1b: HCV clinic visit details—treatment time period Oct 2016 to Nov 2017—Genotype 3 patients. Table S2: HCV clinic visit details—treatment time period Dec 2017 to Dec 2018. Table S3: Odds ratios for not completing treatment. Table S4: Odds ratios for not attending an SVR12 appointment. Table S5: Staff activities. Table S6: Activities, resources and estimated unit costs. Table S7: Estimated unit costs for clinic visits, laboratory tests and medicines. Table S8: Breakdown for fixed costs for HCV clinic overheads. Table S9: Mean per‐patient cost for clinic visits overall and by APRI score. Table S10: Resource use—HCV laboratory investigations. [file JVH-33-0-s001.docx]

Supplementary Materials

**Delivering effective hepatitis C virus treatment in an embedded primary care setting within a tertiary care hospital in Karachi, Pakistan**

**Authors:**

Sabeen Shah^1,2,^*, Nyashadzaishe Mafirakureva^3,4^, Adam Trickey^3^, Aliya Hasnain^5^, Uzma Khan^6,7,8^, Saira Khowaja^6^, Hiba Ashraf^1^, Naila Baig-Ansari^6,9^, Matthew Hickman^3^, Peter Vickerman^3^, Josephine G. Walker^3^, Aaron G. Lim^3,^*

^1^Global Health Directorate, Indus Hospital & Health Network

^2^Department of Family Medicine, Aga Khan University, Pakistan

^3^Population Health Sciences, Bristol Medical School, University of Bristol, Bristol, UK

^4^Health Economics and Decision Science, School of Health & Related Research, University of Sheffield, UK

^5^Department of Medicine, Aga Khan University, Pakistan

^6^Interactive Research and Development (IRD) Global, Singapore

^7^Department of Epidemiology, Biostatistics and Occupational Health, McGill University, Montréal, Canada

^8^TBPeople, Canada

^9^Indus Hospital Research Centre, Indus Hospital & Health Network

*Corresponding author: [aaron.lim@bristol.ac.uk](mailto:aaron.lim@bristol.ac.uk)

*Co-corresponding author: [sshahis80@gmail.com](mailto:sshahis80@gmail.com)

Table of contents

[Referral criteria for the Hepatitis C Control Program 3](#_Toc213432484)

[Investigations 3](#_Toc213432485)

[Treatment Initiation at Hepatitis C clinic 3](#_Toc213432486)

[Treatment Deferral 3](#_Toc213432487)

[Referral to Specialist Clinic 4](#_Toc213432488)

[Treatment regimens 4](#_Toc213432489)

[Time periods 5](#_Toc213432490)

[Study time period 5](#_Toc213432491)

[Electronic database time period 5](#_Toc213432492)

[Statistical analysis 5](#_Toc213432493)

[Study endpoints 5](#_Toc213432494)

[Cascade of care split by treatment period 5](#_Toc213432495)

[Adverse events 5](#_Toc213432496)

[Costing analysis approach 6](#_Toc213432497)

[Identifying resources used 6](#_Toc213432498)

[Measuring resource utilisation 6](#_Toc213432499)

[Valuation of resources 6](#_Toc213432500)

[Supplementary Figures and Tables 8](#_Toc213432501)

[Supplementary Figure S1. Estimated timing of activities. 9](#_Toc213432502)

[Supplementary Figure S2. Mean HCV treatment unit costs by APRI scores. 10](#_Toc213432503)

[Supplementary Figure S3. Mean HCV treatment unit costs by METAVIR stages. 11](#_Toc213432504)

[Supplementary Figure S4. Mean HCV treatment unit costs by treatment duration or reason for stopping treatment. 12](#_Toc213432505)

[Supplementary Table S1a. HCV clinic visit details – treatment time period Oct 2016 to Nov 2017 – Non-Genotype 3 patients 13](#_Toc213432506)

[Supplementary Table S1b. HCV clinic visit details – treatment time period Oct 2016 to Nov 2017 – Genotype 3 patients 14](#_Toc213432507)

[Supplementary Table S2. HCV clinic visit details – treatment time period Dec 2017 to Dec 2018 15](#_Toc213432508)

[Supplementary Table S3. Odds ratios for not completing treatment 16](#_Toc213432509)

[Supplementary Table S4. Odds ratios for not attending an SVR12 appointment. 17](#_Toc213432510)

[Supplementary Table S5. Staff activities. 18](#_Toc213432511)

[Supplementary Table S6. Activities, resources and estimated unit costs. 19](#_Toc213432512)

[Supplementary Table S7. Estimated unit costs for clinic visits, laboratory tests and medicines. 20](#_Toc213432513)

[Supplementary Table S8. Breakdown for fixed costs for HCV clinic overheads. 21](#_Toc213432514)

[Supplementary Table S9. Mean per-patient cost for clinic visits overall and by APRI score. 22](#_Toc213432515)

[Supplementary Table S10. Resource use – HCV laboratory investigations. 23](#_Toc213432516)

Referral criteria for the Hepatitis C Control Program

Only hepatitis C virus (HCV) polymerase chain reaction (PCR) positive patients, indicating active viraemic infection with HCV, were referred for enrolment. Patients could be referred from other Indus Hospital Health Network (IHHN) departments or from other hospitals. It is estimated that around 80-90% patients presenting to clinic were from the Indus Hospital Korangi campus (site of the HCV clinic).

Investigations

Blood tests and liver ultrasound were ordered during first clinic visit to guide subsequent management decisions based on severity/complexity of illness. This was based on APRI, Child Turcotte Pugh (CTP) scoring and comorbid illness profile. From October 2016 to November 2017, blood tests included complete blood count (CBC), aspartate aminotransferase (AST) also known as serum glutamic-oxaloacetic transaminase (SGOT), total bilirubin, serum albumin, prothrombin time, estimated glomerular filtration rate (eGFR), hepatitis B surface antigen (HepBsAg), thyroid stimulating hormone (TSH), Human immunodeficiency virus (HIV) screening, random blood sugar (RBS), Haemoglobin A1c (HbA1c) and genotyping. When clinically indicated pregnancy confirmation test was also ordered. PCR for End of Treatment Response (ETR) and 12 weeks post-treatment completion (SVR12) were conducted to confirm successful treatment response.

Investigation list was optimized during the treatment period December 2017 to December 2018. This was based on data review from October 2016 to November 2017 time period. Investigations with limited value in guiding subsequent management decisions were discontinued such as 1) genotyping due a predominantly genotype 3 treatment cohort, 2) ETR due to high cure rate, 5) TSH due to shift in the choice of medications- 2) HbA1c due shift in choice of medications, and 6) Fibroscan due to logistical and financial barriers and 2) HIV screening due to low seroprevalence.

**See Supplementary Table S1. HCV clinic visit details – treatment time period Oct 2016 to Nov 2017 and Supplementary Table S2. HCV clinic visit details – treatment time period Dec 2017 to Dec 2018**

Treatment Initiation at Hepatitis C clinic

HCV PCR positive patients with APRI score ≥ 0.5, treatment-naive, of age ≥18 to 60 years old, having genotypes 1, 2 or 3, and CTP class A were eligible for treatment initiation through HCV clinic. Eligible patients had to be willing to follow strict contraception for up to 6 months after the end of treatment due to the prescription of ribavirin. Women of childbearing age and health care workers were prioritised for early treatment initiation, regardless of APRI score.

Patients with anaemia (haemoglobin less than 9gm/dl) were assessed for iron deficiency or multi-system involvement. Patients with suspected multi-system involvement were referred to family medicine clinic for detailed evaluation. These patients were reconsidered for treatment initiation once haemoglobin levels normalised.

Treatment Deferral

Limited medication stock and a long patient wait list led to treatment prioritisation for patients with APRI ≥ 0.5. Hence patients with APRI < 0.5 were deferred for treatment and kept on regular 6 monthly monitoring. These patients were assessed for disease progression using APRI score.

Referral to Specialist Clinic

**Gastroenterology**

- - Age less than 18 years
  - Age above 60 years age
  - Any evidence of advanced disease suggestive on clinical examination, biochemical or radiological workup. This included decompensated cirrhosis, patients with CTP class B or C and any sign of hepatocellular carcinoma.
  - Patients with genotypes 4, 5, or 6
  - HepBsAg positive
  - Positive ETR or SVR 12 PCR result (treatment non-responders or failure cases)
  - Patients with a pulse <60 beats/min, on amiodarone or beta blocker
  - Patients not tolerating treatment and requiring discontinuation of treatment due to severe side-effects.

**Referral criteria to infectious disease clinic:** Patients with HIV or tuberculosis infection

All patients referred to the gastroenterology or infectious disease clinics were excluded from the analyses as the processes in speciality clinics were not standardised nor captured on a regular basis by the HCV Control Program team.

Treatment regimens

Treatment start dates were divided into two periods to coincide with changes in HCV treatment program leadership. From October 2016 to November 2017 program operations were managed under umbrella of Indus Hospital Research Centre, whereas during December 2017 to December 2018 operations were aligned under the department of Global Health Directorate. This shift was to line up future expansion of the treatment program with Primary Care Program and other community based public health programs operated from the platform of Global Health Directorate.

First 14 months of program initiation, regimen prescribed was based on genotype result.

- Genotype 3 patients were prescribed sofosbuvir (400 mg/day) and weight-based ribavirin (1000 to 1200 mg/day) for 24 weeks
- Genotype 1 patients prescribed Sofosbuvir (400mg/day), weight-based Ribavirin (1000 to 1200 mg/day) and Pegylated interferon (180 μg/week) for 12 weeks
- Genotype 2 patients were prescribed Sofosbuvir 400mg and weight-based Ribavirin (1000 to 1200 mg/day for 12 weeks.

A dedicated clinical team (one nurse and a doctor) was trained and assigned to provided care in a designated clinic space (HCV clinic).

Major shifts in clinical protocol from December 2017 onwards were 1) discontinuation of genotyping and ETR PCR test, 2) prescription of sofosbuvir (400 mg/day) and daclatasvir (60mg/day) for 12 weeks to all patients except those with compensated cirrhosis, weight based ribavirin (1000 to 1200 mg/day) was added and treated for total duration of 24 weeks 3) reduction in number of pre-treatment initiation visits from three to two and 4) training of all family medicine nurses and doctors to provide algorithm-based care, however the responsibility of running the clinic by this trained cohort of family medicine nurses and doctors was after December 2018.

Time periods

Study time period

The pilot HCV Control Programme took place from October 2016 to December 2018.

Electronic database time period

All patient-level data covered the observation period plus nine months afterwards to capture follow-up patient outcomes and resource use, i.e. from October 2016 to September 2019.

Statistical analysis

Study endpoints

The endpoints considered were 1) completed treatment, had PCR test for sustained virological response at 12 weeks (SVR12), 2) incomplete treatment, and 3) complete treatment but loss to follow-up (LTFU) Pre SVR 12. The reason for incomplete treatment was recorded with loss to follow-up being defined as those that did not attend their treatment completion visit. Loss to follow-up pre-SVR12 visit was defined as those that did not attend their SVR12 visit. Adverse events were self-reported and recorded by the clinician. Overall, there were few missing data, with previous surgery being the variable most affected as 170 patients (13.2%) were missing this information.

Cascade of care split by treatment period

Looking at the outcomes split by treatment start date period, 229 (17.8%) started between October 2016 and November 2017, whilst 1059 (82.2%) started between December 2017 and December 2018. Of the 229 starting during the first period (October 2016 to November 2017), 9 (3.9%) did not complete their treatment, with 7 (77.7%) of these LTFU and 2 stopping treatment. Of the 220 that completed treatment, 11 (5.0%) did not attend their SVR12 follow-up appointment, with 204/209 (97.6%) that did attend having an undetectable viral load. For the 1059 patients starting during the second period (December 2017 to December 2018), 79 (7.4%) did not complete their treatment. A total of 980 patients completed treatment, of whom 305 (31.1%) did not attend their SVR12 follow-up appointment. Of those attending their SVR12 appointment, 666/675 (98.7%) had an undetectable viral load.

Adverse events

In total, 50 people (3.9%) had adverse events, with 28 people reporting one adverse event, 14 people reporting two adverse events, and 8 people reporting three adverse events, giving a total of 80 adverse events. Of these, 15 were haematological, 10 were gastrointestinal, 19 were neurological, 16 were musculoskeletal, and 8 were classified as other (rash, burning sensation on scalp, dyspnoea [x2], fever, palpitations, chest pain at rest, transient ischemic attack).

Costing analysis approach

Identifying resources used

A detailed review of the HCV Control Program, its clinical protocol and recommendations, and interviews with key technical staff (program manager, technical advisor, research associate, nurses, doctors) involved in the planning, implementation and coordination of the program was performed to identify all the activities and resources utilised in the treatment of patients with chronic HCV at IHHN.

The main activities in the program included baseline medical assessment for treatment eligibility, baseline laboratory work up, patient counselling/education, hepatitis B vaccination, direct acting antiviral (DAA) treatment initiation, treatment follow-up, treatment monitoring laboratory tests, routine management of medical problems, on-treatment referrals to other medical services (e.g. gastroenterologist), post-treatment follow up, ETR and SVR12 assessment.

The resources identified included patient-facing staff time (receptionist, porter, nurse, phlebotomist, doctor, pharmacist), laboratory tests (HCV PCR, blood tests, HepBsAg, HIV), DAA medicines, and overheads. Overheads included administrative overheads, indirect costs (not directly related to patient care), support staff (not directly seeing patients), coordination staff (program manager), training, medical and laboratory supplies, non-medical supplies.

Measuring resource utilisation

Primary data were collected on the exact number and type of resources consumed during the treatment program. Detailed patient-level data, including the number and type of clinic visits, clinical examinations, laboratory investigations, treatment regimens, and treatment outcomes were extracted from data collected during the study using an electronic database (October 2016 – September 2019). The amount of time spent by staff providing services in the program was derived from an internal process flow for the HCV clinic done as part of a separate Time-Driven Activity-Based Costing (Supplementary Figure S1). Overhead and administrative costs associated with the program were allocated using the step-down costing approach. For example, staff numbers in each department were used to allocate management and administrative costs, service statistics were used to allocate shared recurrent resources.

Valuation of resources

Valuation of the resources used in the treatment program was based on the Indus Hospital’s HCV program’s financial records and information provided by program staff (finance, logistics, and pharmacy staff). The most up-to-date unit prices/costs were applied to patient-level data on resource use to estimate the costs of treating each patient. Unit costs for supplies and consumables, including laboratory tests, were obtained from the Indus Hospital’s HCV programs’ financial records and supplemented with interviews with key personnel (finance, logistics, and HCV program manager). Unit costs for valuing staff time were estimated based on staff salaries information provided by the Indus Hospital. Valuation of the DAAs was based on the prices paid for the medicines by the Indus Hospital at the time of purchase. Building costs (rentals) were provided as part of the Indus Hospital’s HCV programs’ financial records.

All unit prices were gathered in the local currency (Pakistani Rupee), adjusted for inflation to 2019 prices using the Consumer Price Indices for Pakistan obtained from IMF World Economic Outlook database (International monetary fund, 2022) and then converted to the USD currency using the 2019 average market-based exchange rate (1 USD = 136 Pakistani Rupee). The per unit overhead costs for each activity were estimated by dividing the annual total cost for the activity by the annual total number of units of output (for example, number of patient visits or patients). The cost of each activity is the sum of the costs for all the resources used in executing that activity, i.e. labour, consumables and overheads. The activity costs were multiplied by the number of times a patient received each activity and summed to give an estimate of the total cost per patient. The total costs comprised of the following categories: HCV-related visit costs, HCV treatment related laboratory costs and DAA costs.

*HCV visit costs:* HCV-related visits comprised of all visits by patients in preparation for, during and after treatment. These included baseline medical assessments, treatment initiation, on-treatment follow up, end of treatment, post-treatment follow-up and SVR assessment visits. Each visit cost included the cost of staff time specific to the visit (based on the process flow for the HCV clinic) and space/materials for the HCV clinic. The visit cost incorporated recurrent costs (support personnel costs, medical supplies, non-medical supplies, medical services and diagnostic charges, utilities and bills) and indirect costs (buildings, vehicles, construction and rehabilitation, and furniture).

*HCV treatment-related laboratory costs:* Laboratory costs included all laboratory tests and investigations performed for each patient in preparation for, during and after treatment according to the Indus Hospital’s HCV Control Program’s clinical protocol obtained from Indus Hospital HCV program’s financial records.

*DAA medicine costs:* Unit costs for DAAs were determined from the Indus Hospital HCV program’s financial records. DAA costs for each patient were calculated based on the patients-specific treatment regimen and the length of treatment obtained from the electronic database.

Supplementary Figures and Tables

| **(A)** |
| --- |
| **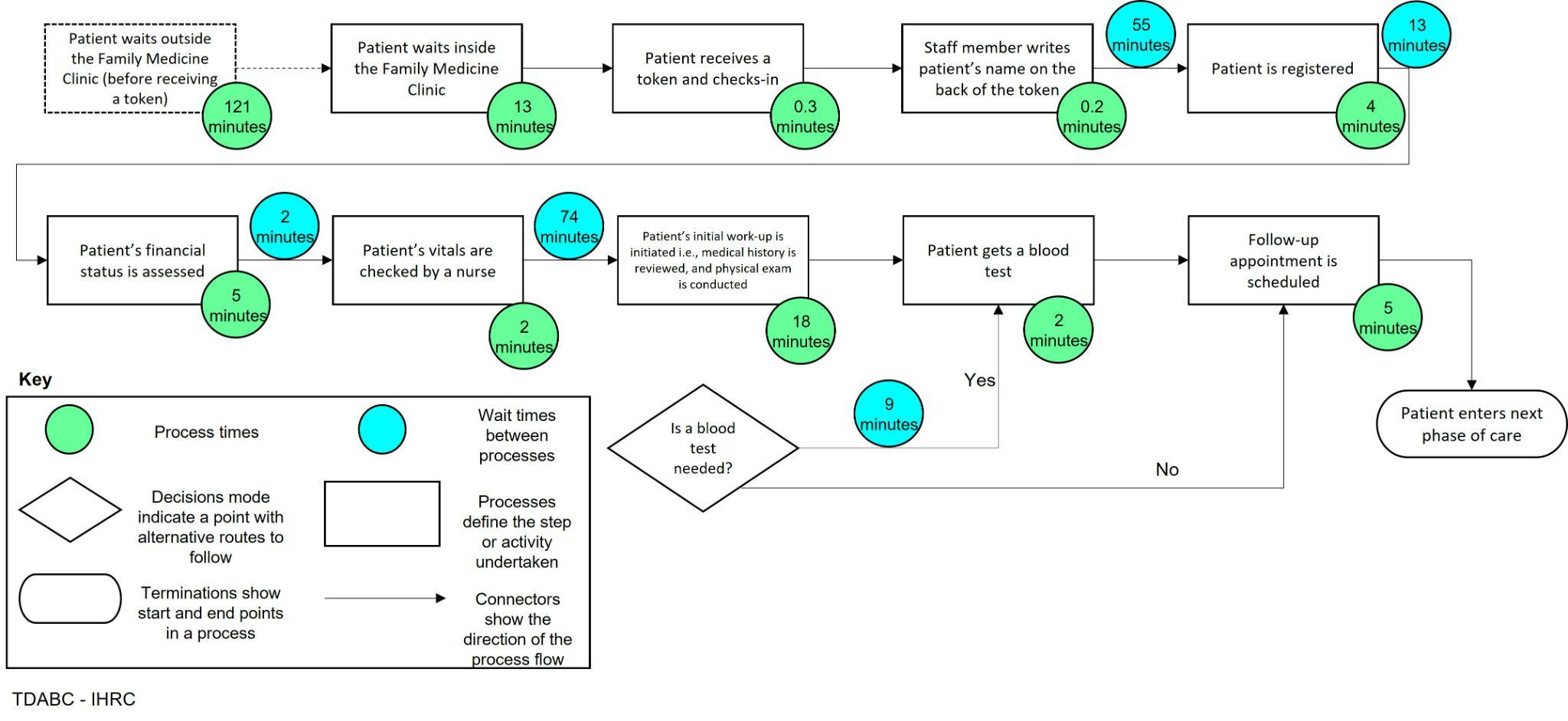** |
| **B)** |
| 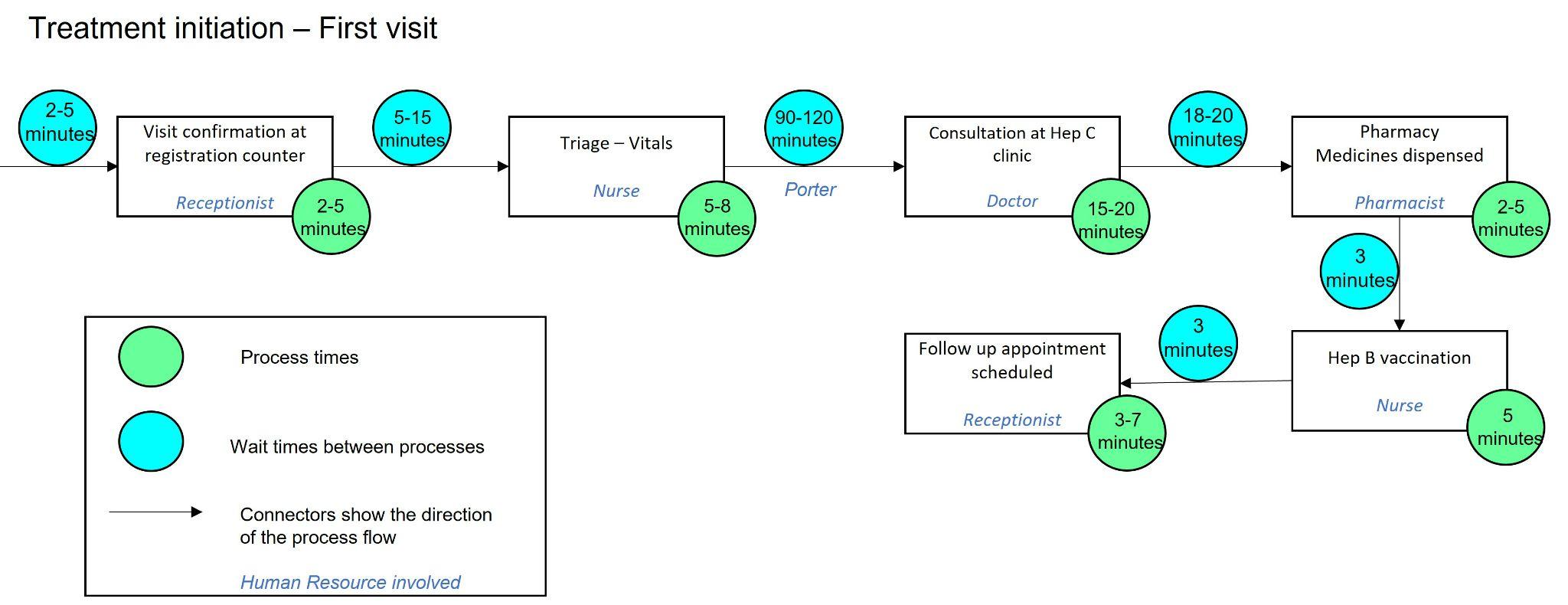 |

| **(C)**  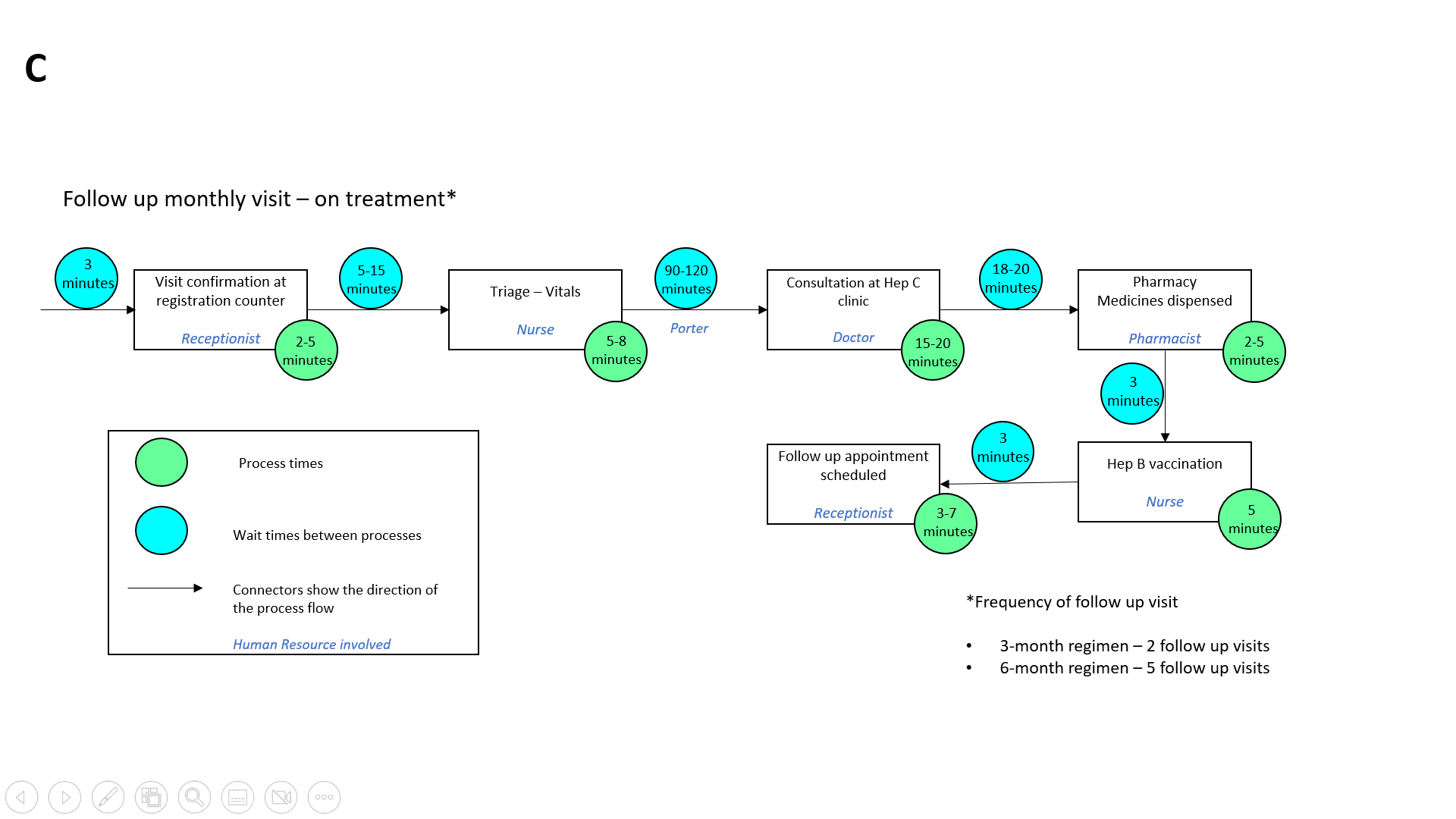 |
| --- |
|  |
| **(D)**  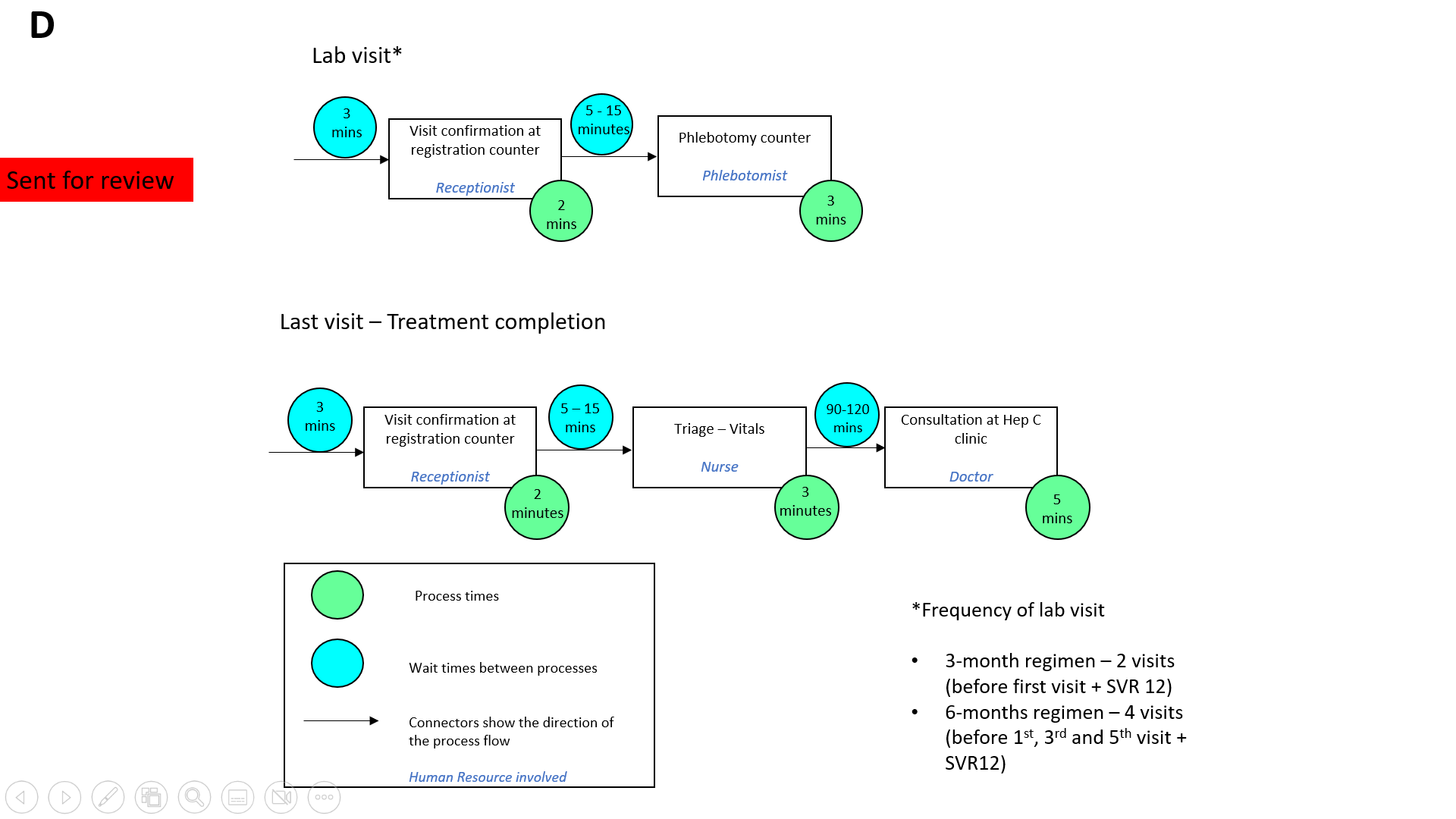 |
|  |

Supplementary Figure S1. Estimated timing of activities.

Estimates of time taken to undertake various activities using a Time-Driven Activity-Based approach. The flow charts show the various pathways for a patient in the hepatitis C treatment program: (A) registration visit; (B) during treatment initiation; (C) during monthly visits on treatment; and (D) for lab visits.

| **(A)** |
| --- |
| **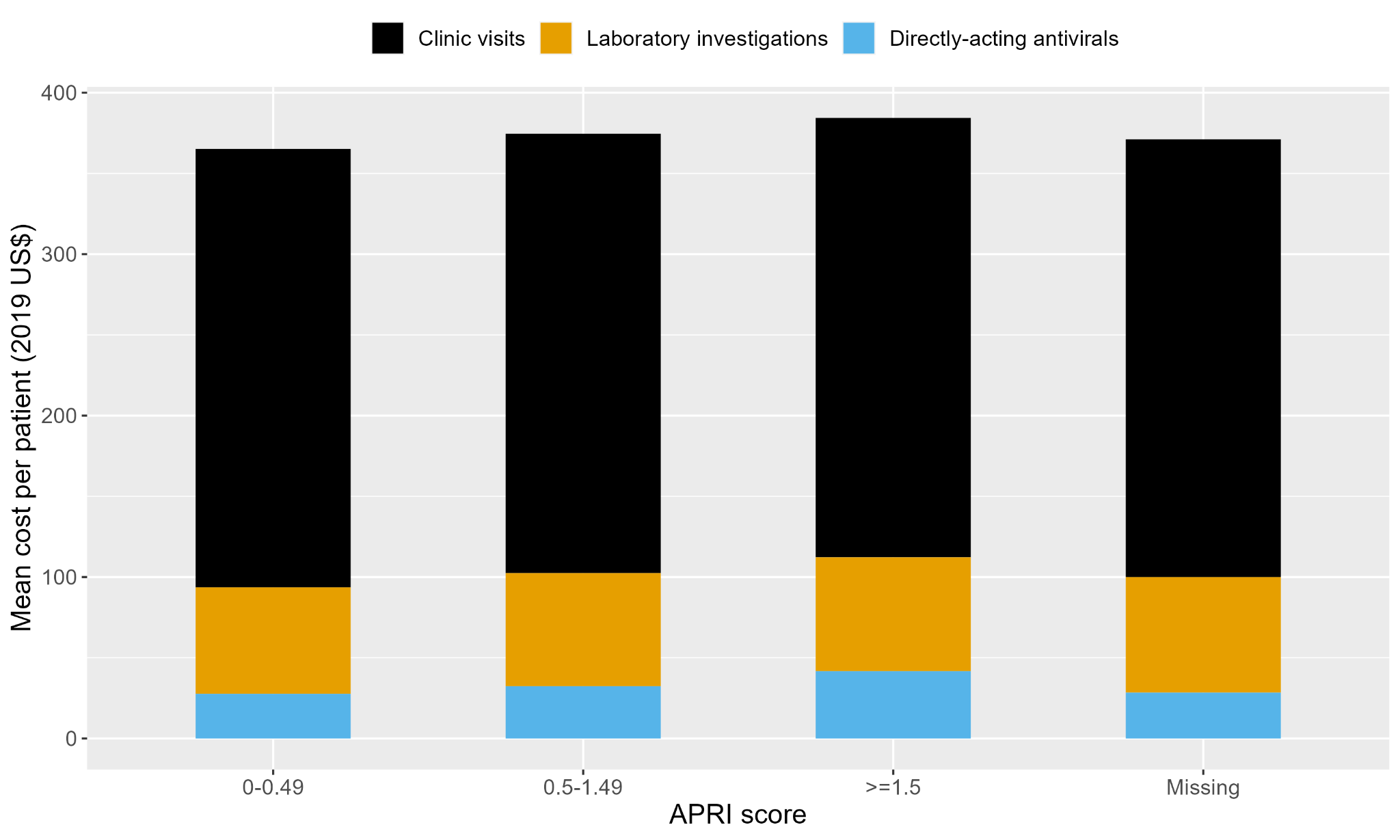** |
| **(B)** |
| 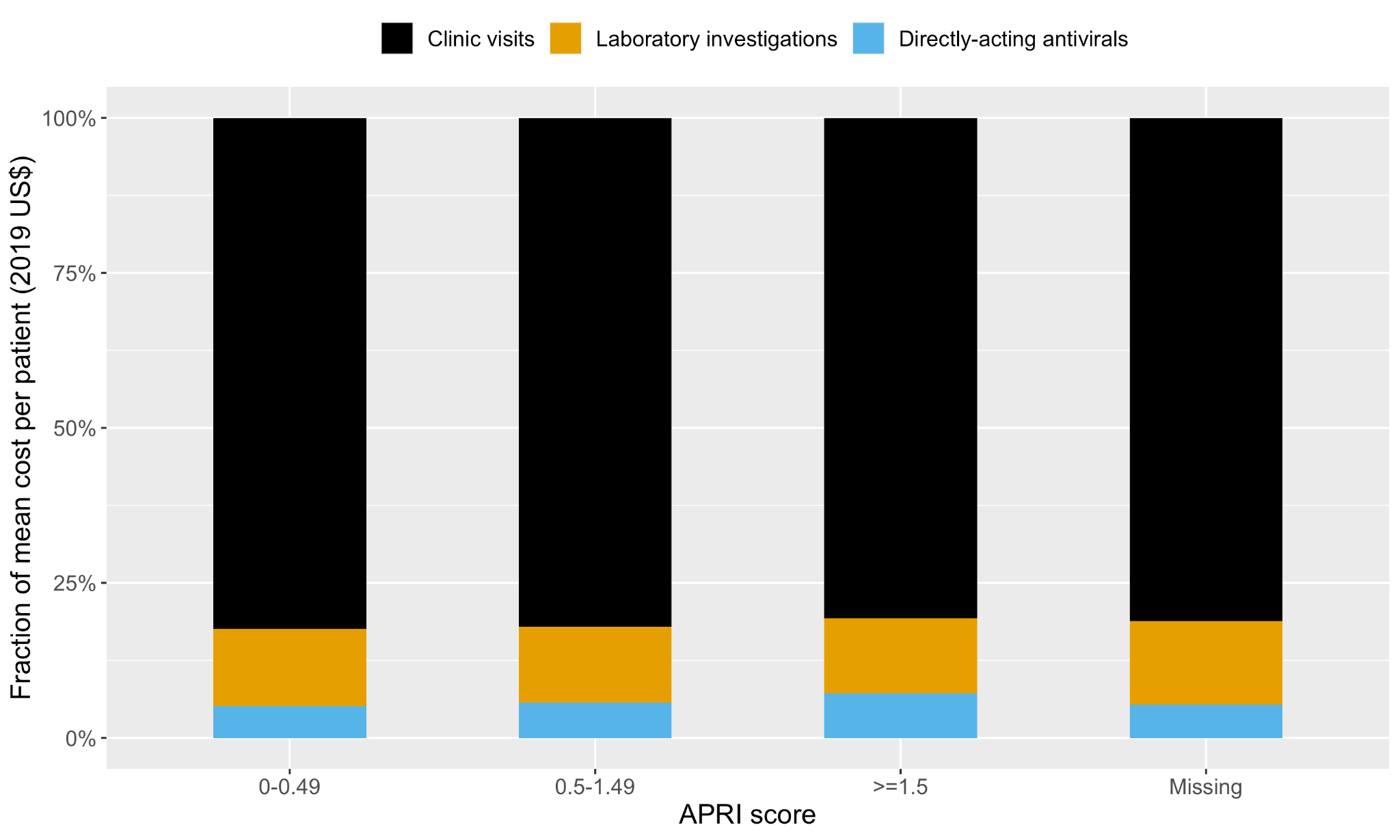 |

Supplementary Figure S2. Mean HCV treatment unit costs by APRI scores.

The mean HCV treatment costs per patient by aspartate aminotransferase to platelet ratio index (APRI) scores with respect to (A) distribution and (B) fraction of total costs.

| **(A)** |
| --- |
| 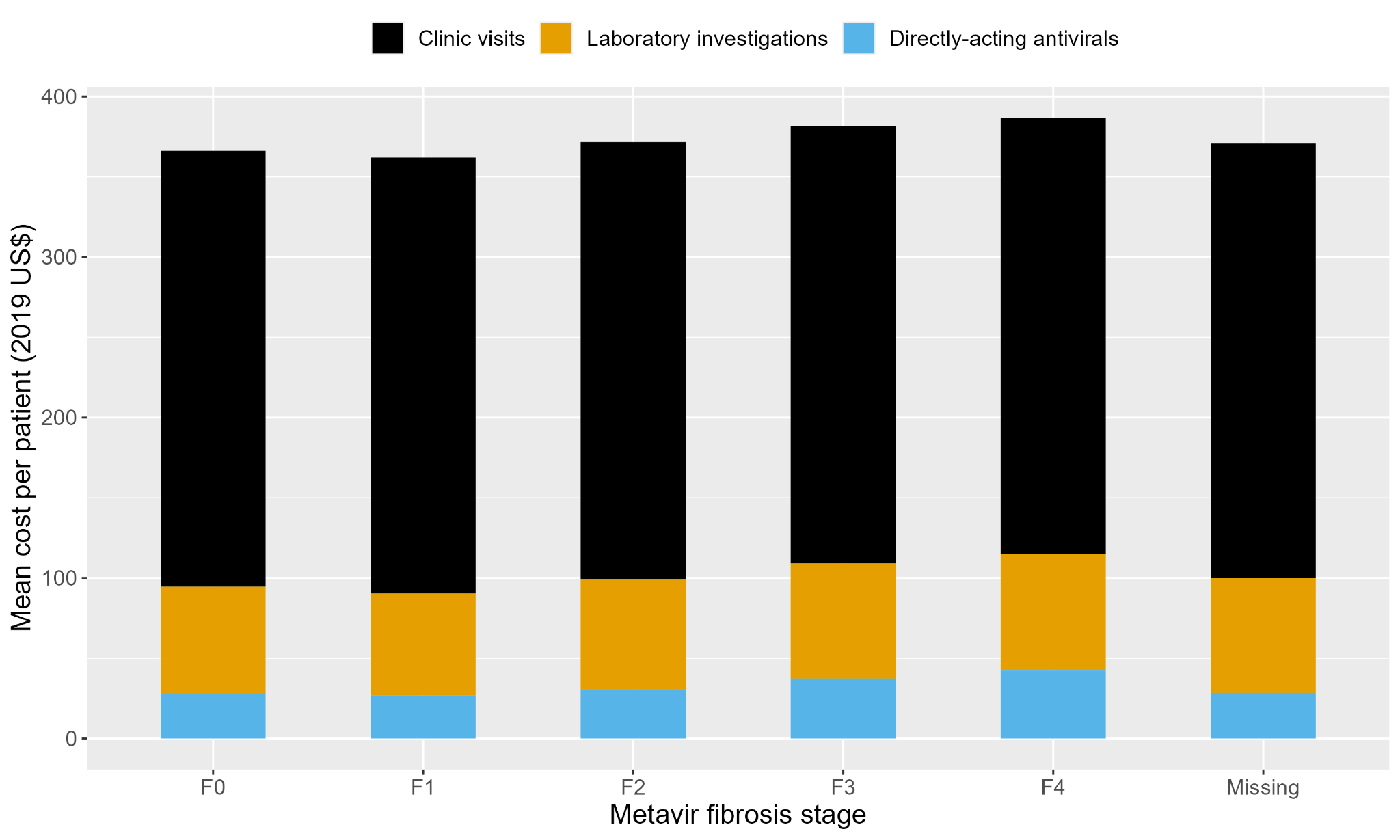 |
| **(B)** |
| 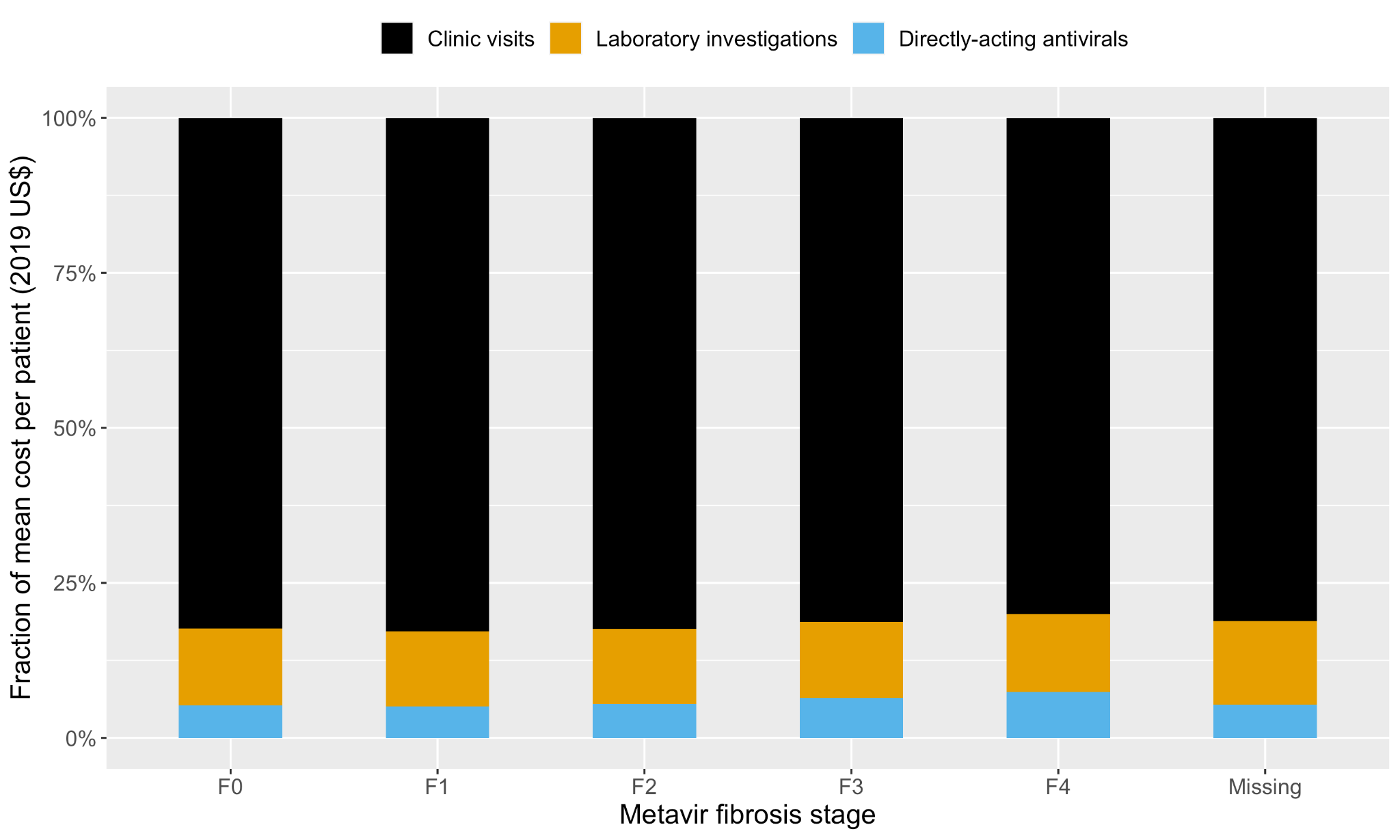 |

Supplementary Figure S3. Mean HCV treatment unit costs by METAVIR stages.

The mean HCV treatment costs per patient by METAVIR fibrosis stages with respect to (A) distribution and (B) fraction of total costs.

| **(A)** |
| --- |
| 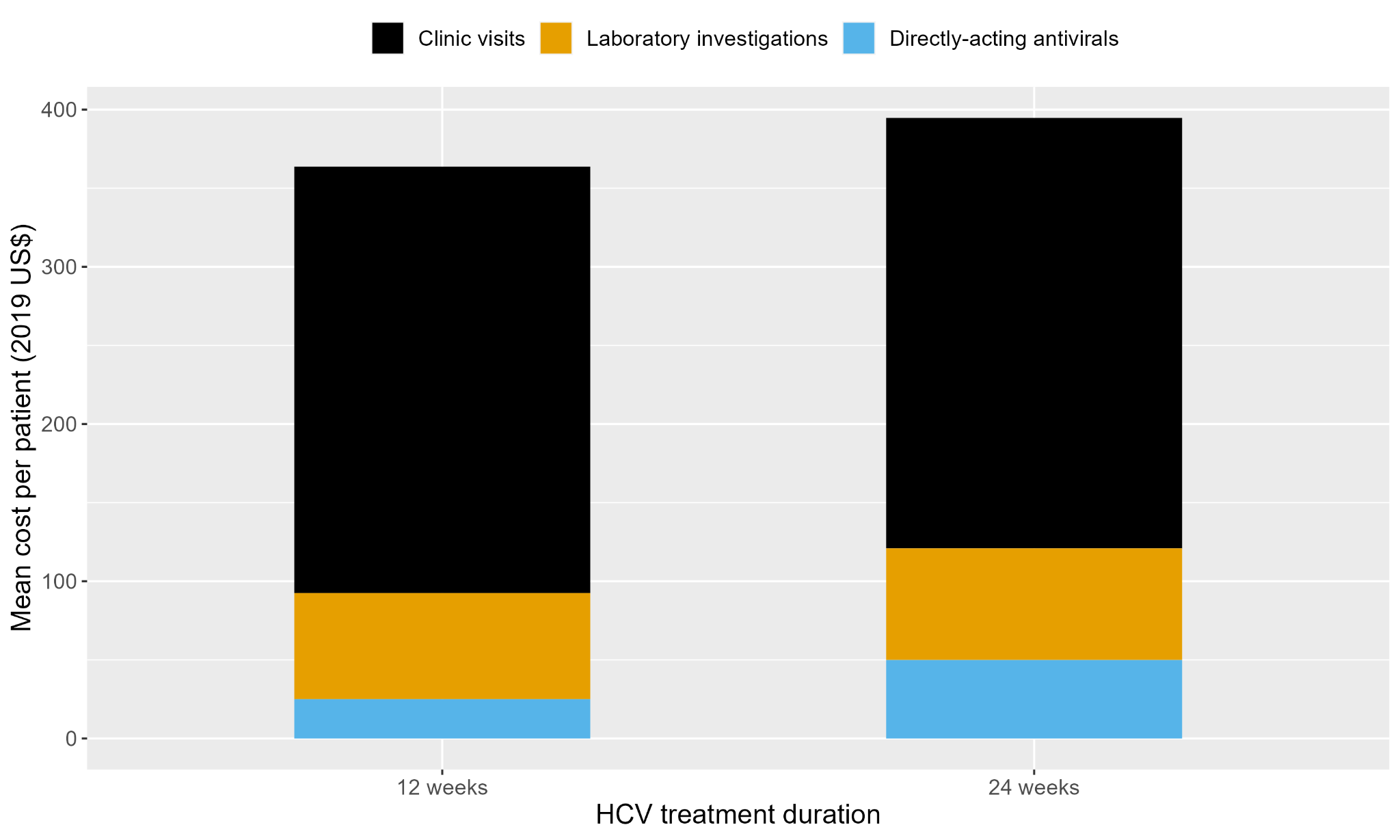 |
| **(B)** |
| 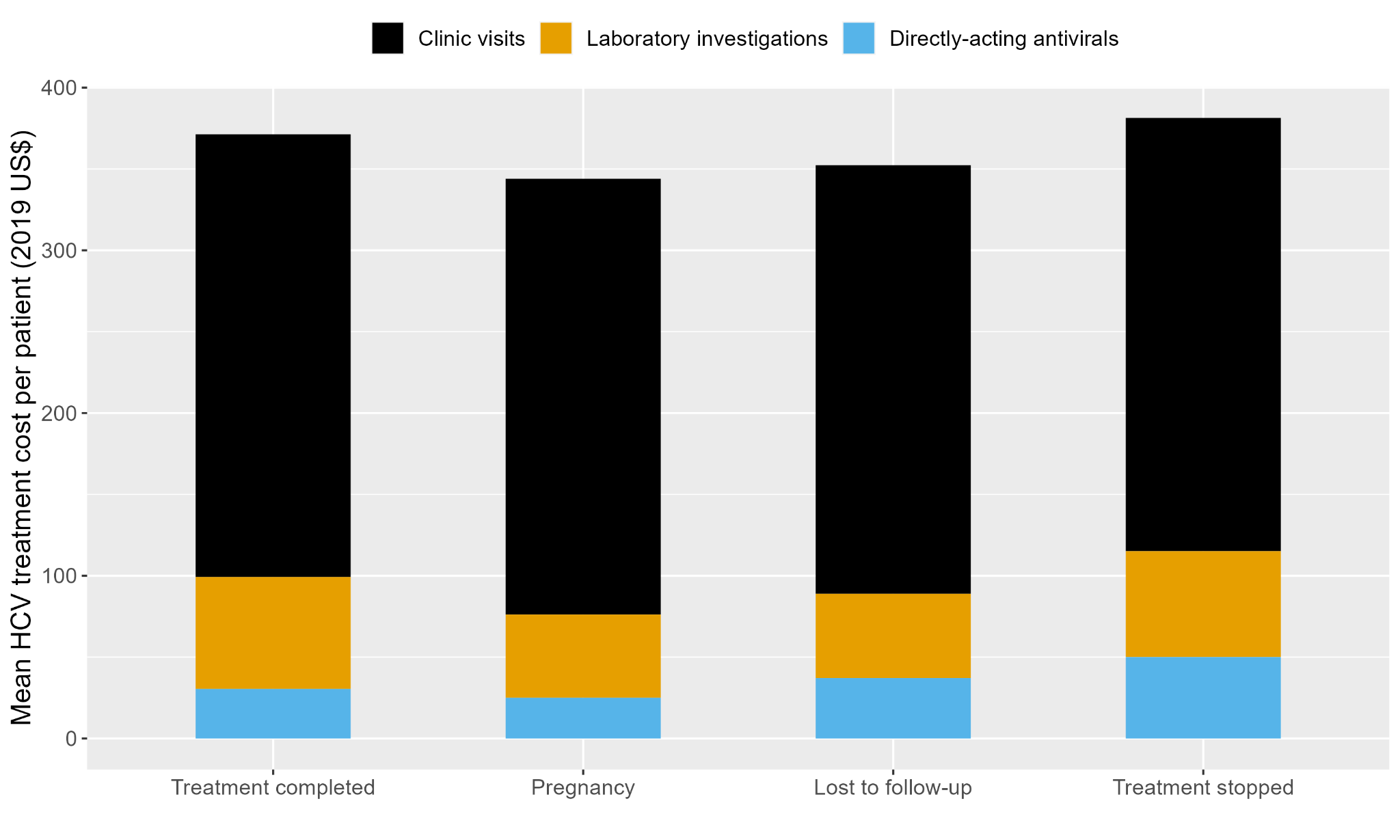 |

Supplementary Figure S4. Mean HCV treatment unit costs by treatment duration or reason for stopping treatment.

The mean HCV treatment costs per patient by (A) treatment duration or (B) reason for stopping treatment.

Supplementary Table S1a. HCV clinic visit details – treatment time period Oct 2016 to Nov 2017 – Non-Genotype 3 patients

|  | **Treatment time period**  **October 2016 to November 2017** | **Baseline**  **medical assessment** | **Wk**  **0** | **Wk**  **4** | **Wk**  **8** | **Wk**  **12** | **Wk 12**  **post treatment completion** |
| --- | --- | --- | --- | --- | --- | --- | --- |
| **1** | **Doctor consultation** | X | X | X | X | X |  |
| **2** | **Medicine disbursement** |  |  |  |  |  |  |
|  | GT 1 Sofosbuvir +Ribavirin + Peg IFN – 12 weeks |  | X | X | X |  |  |
|  | GT 2 sofosbuvir + ribavirin – 12 weeks |  | X | X | X |  |  |
|  | Assess for drug adherence and adverse effects |  | X | X | X | X |  |
| **3** | **Investigations** |  |  |  |  |  |  |
| 3.1 | Complete blood count | X |  | X |  | X |  |
| 3.2 | Liver function test | X |  |  |  |  |  |
| 3.3 | Serum Creatinine | X |  |  |  |  |  |
| 3.4 | Serum Albumin | X |  |  |  |  |  |
| 3.5 | PT/ INR* | X |  |  |  |  |  |
| 3.6 | TSH* | X |  |  |  |  |  |
| 3.7 | HbA1c * (Random blood sugar if diagnosed case of diabetes mellitus) | X |  |  |  |  |  |
| 3.8 | Urine pregnancy test | X |  |  |  |  |  |
| 3.9 | HCV PCR* | X |  |  |  | X | X |
| 3.10 | HCV genotyping | X |  |  |  |  |  |
| 3.11 | HBVsAg * (ELISA) - (if vaccinated HBV Ab) | X |  |  |  |  |  |
| 3.12 | HIV* screening | X |  |  |  |  |  |
| 3.13 | Ultrasound liver | X |  |  |  |  |  |
| 3.14 | Elastography (Fibroscan) – for patients with APRI*score > 0.5 | X |  |  |  |  |  |

*Abbreviations: Ab: antibody, APRI: aspartate aminotransferase to platelet ratio index, ELISA: enzyme-linked immunosorbent assay, GT: genotype, HbA1c: hemoglobin A1c, HBVsAg: hepatitis B virus surface antigen, HCV: hepatitis C virus, HIV: human immunodeficiency virus, INR: international normalised ratio, PCR: polymerase chain reaction, Peg INF: pegylated interferon, PT: prothrombin time, TSH: thryoid stimulating hormone

Supplementary Table S1b. HCV clinic visit details – treatment time period Oct 2016 to Nov 2017 – Genotype 3 patients

|  | **Treatment time period**  **October 2016 to November 2017** | **Baseline**  **medical assessment** | **Wk**  **0** | **Wk**  **4** | **Wk**  **8** | **Wk**  **12** | **Wk**  **16** | **Wk**  **20** | **Wk 24** | **Wk 12**  **post treatment completion** |
| --- | --- | --- | --- | --- | --- | --- | --- | --- | --- | --- |
| **1** | **Doctor consultation** | X | X | X | X | X | X | X | X |  |
| **2** | **Medicine disbursement** |  |  |  |  |  |  |  |  |  |
|  | Sofosbuvir + Ribavirin – 24 weeks |  | X | X | X | X | X | X | X |  |
|  | Assess for drug adherence and adverse effects |  | X | X | X | X | X | X | X |  |
| **3** | **Investigations** |  |  |  |  |  |  |  |  |  |
| 3.1 | Complete blood count | X |  | X |  | X |  |  | X |  |
| 3.2 | Liver function test | X |  |  |  |  |  |  |  |  |
| 3.3 | Serum Creatinine | X |  |  |  |  |  |  |  |  |
| 3.4 | Serum Albumin | X |  |  |  |  |  |  |  |  |
| 3.5 | PT/ INR* | X |  |  |  |  |  |  |  |  |
| 3.6 | HbA1c * (Random blood sugar if diagnosed case of diabetes mellitus) | X |  |  |  |  |  |  |  |  |
| 3.7 | Urine pregnancy test | X |  |  |  |  |  |  |  |  |
| 3.8 | HCV PCR* | X |  |  |  |  |  |  | X | X |
| 3.9 | HCV genotyping | X |  |  |  |  |  |  |  |  |
| 3.10 | HBVsAg * (ELISA) - (if vaccinated HBV Ab) | X |  |  |  |  |  |  |  |  |
| 3.11 | HIV* screening | X |  |  |  |  |  |  |  |  |
| 3.12 | Ultrasound liver | X |  |  |  |  |  |  |  |  |
| 3.13 | Elastography (Fibroscan) – for patients with APRI*score > 0.5 | X |  |  |  |  |  |  |  |  |

*Abbreviations: Ab: antibody, APRI: aspartate aminotransferase to platelet ratio index, ELISA: enzyme-linked immunosorbent assay, GT: genotype, HbA1c: hemoglobin A1c, HBVsAg: hepatitis B virus surface antigen, HCV: hepatitis C virus, HIV: human immunodeficiency virus, INR: international normalised ratio, PCR: polymerase chain reaction, Peg INF: pegylated interferon, PT: prothrombin time

Supplementary Table S2. HCV clinic visit details – treatment time period Dec 2017 to Dec 2018

|  | **Treatment time period**  **December 2017 to December 2018** | **Baseline medical assessment** | **Wk**  **0** | **Wk**  **4** | **Wk 8** | **Wk**  **12** | **Wk 16** | **Wk 20** | **Wk**  **24** | **Wk 12**  **post treatment completion** |
| --- | --- | --- | --- | --- | --- | --- | --- | --- | --- | --- |
| **1** | **Doctor consultation** | X | X | X | X | X | X | X | X | X |
| **2** | **Medicine disbursement** |  |  |  |  |  |  |  |  |  |
|  | Patients with no cirrhosis  Sofosbuvir + daclatasvir - 12 weeks |  | X | X | X |  |  |  |  |  |
|  | Patients with compensated cirrhosis  Sofosbuvir + daclatasvir and weight-based ribavirin – 24 weeks |  | X | X | X | X | X | X |  |  |
|  | Assess for drug adherence and adverse effects |  | X | X | X | X | X | X |  |  |
| **3** | **Investigations** |  |  |  |  |  |  |  |  |  |
| 3.1 | Complete blood picture | X |  | X† |  | X† |  |  | X† |  |
| 3.2 | Liver function test | X |  |  |  |  |  |  |  |  |
| 3.3 | Serum Creatinine | X |  |  |  |  |  |  |  |  |
| 3.4 | Serum Albumin | X |  |  |  |  |  |  |  |  |
| 3.5 | PT/ INR | X |  |  |  |  |  |  |  |  |
| 3.6 | Urine pregnancy test | X |  |  |  |  |  |  |  |  |
| 3.7 | HCV PCR | X |  |  |  |  |  |  |  | X |
| 3.8 | HBVsAg (ELISA) (if vaccinated HBV Ab) | X |  |  |  |  |  |  |  |  |
| 3.9 | Ultrasound liver | X |  |  |  |  |  |  |  |  |

†Patients on ribavirin

*Abbreviations: Ab: antibody. ELISA: enzyme-linked immunosorbent assay, HBVsAg: hepatitis B virus surface antigen, HCV: hepatitis C virus, INR: international normalised ratio, PCR: polymerase chain reaction, PT: prothrombin time

Supplementary Table S3. Odds ratios for not completing treatment

Odds ratios for not completing treatment among N=1288 that started treatment.

|  | **Odds ratios (95% confidence intervals)** | | | |
| --- | --- | --- | --- | --- |
|  | **Univariable** | **P-value** | **Multivariable** | **P-value** |
| Treatment start period |  |  |  |  |
| Oct 2016 – Nov 2017 | 1 |  | 1 |  |
| Dec 2017 – Dec 2018 | 1.97 (0.97-3.99) | 0.059 | 5.84 (1.07-31.92) | 0.042 |
| Regimen prescribed |  |  |  |  |
| SOF/DAC | 1 |  | 1 |  |
| SOF/RBV | 0.61 (0.28, 1.29) | 0.196 | 1.14 (0.23-5.64) | 0.871 |
| SOF/DAC/RBV | 2.41 (1.45, 4.02) | 0.001 | 0.98 (0.32-2.98) | 0.970 |
| SOF/RBV/Peg-INF | 4.42 (0.90, 21.78) | 0.068 | 10.01 (0.98-102.24) | 0.052 |
| Treatment duration |  |  |  |  |
| 12 weeks | 1 |  | 1 |  |
| 24 weeks | 1.53 (0.98, 2.38) | 0.061 | 2.47 (0.84-7.25) | 0.101 |
| APRI score |  |  |  |  |
| 0 – 0.49 | 1 |  | 1 |  |
| 0.5 – 1.49 | 0.95 (0.57, 1.59) | 0.855 | 0.94 (0.54-1.63) | 0.809 |
| ≥1.5 | 2.00 (1.08, 3.70) | 0.028 | 1.36 (0.64-2.92) | 0.426 |
| Missing | 1.30 (0.59, 2.87) | 0.518 | 1.23 (0.54-2.81) | 0.628 |
| Age (years) |  |  |  |  |
| 0 – 29 | 1 |  | 1 |  |
| 30 – 49 | 0.59 (0.35, 1.02) | 0.059 | 0.47 (0.26-0.83) | 0.009 |
| ≥50 | 0.77 (0.42, 1.40) | 0.397 | 0.48 (0.24-0.93) | 0.030 |
| Sex |  |  |  |  |
| Male | 1 |  | 1 |  |
| Female | 1.20 (0.76, 1.90) | 0.439 | 1.33 (0.78-2.25) | 0.290 |
| Schooling |  |  |  |  |
| None | 1 |  | 1 |  |
| Been to school | 0.84 (0.53, 1.34) | 0.477 | 0.92 (0.55-1.52) | 0.735 |
| Missing | 1.34 (0.66, 2.73) | 0.419 | 6.71 (0.89-50.86) | 0.066 |
| Previous HCV treatment |  |  |  |  |
| No | 1 |  | 1 |  |
| Yes | 1.59 (0.84, 3.04) | 0.157 | 1.22 (0.98-2.70) | 0.584 |
| Missing | 1.27 (0.64, 2.54) | 0.497 | 2.10 (0.03-147.50) | 0.732 |
| Any comorbidity |  |  |  |  |
| No | 1 |  | 1 |  |
| Yes | 1.68 (1.04, 2.71) | 0.034 | 1.63 (0.98-2.70) | 0.058 |
| Missing | 1.17 (0.58, 2.37) | 0.661 | 0.11 (0.00-10.67) | 0.342 |
| Previous surgery |  |  |  |  |
| No | 1 |  | 1 |  |
| Yes | 0.86 (0.54, 1.37) | 0.533 | 0.78 (0.47-1.30) | 0.343 |
| Missing | 0.87 (0.44, 1.73) | 0.699 | NA | NA |
| Substance use addiction |  |  |  |  |
| No | 1 |  | 1 |  |
| Yes | 1.11 (0.65, 1.90) | 0.703 | 1.47 (0.81-2.66) | 0.203 |
| Missing | 1.02 (0.52, 1.98) | 0.960 | NA | NA |

Abbreviations: APRI, Aspartate aminotransferase to Platelet Ratio Index; SVR, sustained virological response; SOF, sofosbuvir; DAC, daclatasvir; RBV, ribavirin; Peg-INF, pegylated interferon

Supplementary Table S4. Odds ratios for not attending an SVR12 appointment.

Odds ratios for not attending an SVR12 appointment among N=1200 that completed treatment.

|  | **Odds ratios (95% confidence intervals)** | | | |
| --- | --- | --- | --- | --- |
|  | **Univariable** | **P-value** | **Multivariable** | **P-value** |
| Treatment start period |  |  |  |  |
| Oct 2016 – Nov 2017 | 1 |  | 1 |  |
| Dec 2017 – Dec 2018 | 8.59 (4.61-15.98) | <0.001 | 23.23 (5.97-90.46) | <0.001 |
| Regimen prescribed |  |  |  |  |
| SOF/DAC | 1 |  | 1 |  |
| SOF/RBV | 0.17 (0.10, 0.31) | <0.001 | 0.93 (0.31-2.80) | 0.894 |
| SOF/DAC/RBV | 1.90 (1.34, 2.71) | <0.001 | 1.00 (0.42-2.35) | 0.998 |
| SOF/RBV/Peg-INF | 0.42 (0.05, 3.53) | 0.427 | 3.41 (0.29-40.21) | 0.330 |
| Treatment duration |  |  |  |  |
| 12 weeks | 1 |  | 1 |  |
| 24 weeks | 0.77 (0.58, 1.03) | 0.079 | 3.46 (1.51-7.93) | 0.003 |
| APRI score |  |  |  |  |
| 0 – 0.49 | 1 |  | 1 |  |
| 0.5 – 1.49 | 0.77 (0.58, 1.03) | 0.078 | 0.69 (0.50-0.96) | 0.029 |
| ≥1.5 | 0.79 (0.50, 1.24) | 0.305 | 0.44 (0.24-0.78) | 0.005 |
| Missing | 0.97 (0.60, 1.58) | 0.903 | 0.66 (0.39-1.11) | 0.118 |
| Age (years) |  |  |  |  |
| 0 – 29 | 1 |  | 1 |  |
| 30 – 49 | 1.28 (0.89, 1.83) | 0.182 | 1.21 (0.82-1.79) | 0.330 |
| ≥50 | 1.08 (0.72, 1.62) | 0.713 | 0.87 (0.55-1.36) | 0.532 |
| Sex |  |  |  |  |
| Male | 1 |  | 1 |  |
| Female | 0.76 (0.59, 0.99) | 0.045 | 0.77 (0.56-1.05) | 0.097 |
| Schooling |  |  |  |  |
| None | 1 |  | 1 |  |
| Been to school | 0.88 (0.66, 1.16) | 0.347 | 0.86 (0.63-1.17) | 0.332 |
| Missing | 1.99 (1.27, 3.08) | 0.002 | 2.22 (0.62-7.95) | 0.220 |
| Previous HCV treatment |  |  |  |  |
| No | 1 |  | 1 |  |
| Yes | 1.12 (0.72, 1.75) | 0.603 | 0.72 (0.44-1.18) | 0.193 |
| Missing | 1.93 (1.29, 2.87) | 0.001 | 0.62 (0.13-2.97) | 0.550 |
| Any comorbidity |  |  |  |  |
| No | 1 |  | 1 |  |
| Yes | 1.16 (0.85, 1.58) | 0.359 | 1.16 (0.83-1.62) | 0.390 |
| Missing | 1.79 (1.22, 2.63) | 0.003 | 0.55 (0.14-2.28) | 0.413 |
| Previous surgery |  |  |  |  |
| No | 1 |  | 1 |  |
| Yes | 0.69 (0.52, 0.92) | 0.012 | 0.74 (0.54-1.01) | 0.057 |
| Missing | 1.57 (1.09, 2.28) | 0.017 | 2.50 (0.56-11.14) | 0.231 |
| Substance use addiction |  |  |  |  |
| No | 1 |  | 1 |  |
| Yes | 0.96 (0.69, 1.35) | 0.833 | 1.21 (0.83-1.79) | 0.322 |
| Missing | 1.77 (1.23, 2.56) | 0.002 | 0.70 (0.12-4.21) | 0.694 |

Abbreviations: APRI, Aspartate aminotransferase to Platelet Ratio Index; SVR, sustained virological response; SOF, sofosbuvir; DAC, daclatasvir; RBV, ribavirin; Peg-INF, pegylated interferon

Supplementary Table S5. Staff activities.

Staff types and staff times for the different activities in the Indus Hospital HCV treatment programme.

|  |  | **Activity time (minutes)** | | |
| --- | --- | --- | --- | --- |
| **Activity** | **Personnel** | **Mean** | **Low** | **High** |
| **Pre-treatment assessment** |  |  |  |  |
| Patient receives token | Porter | 0.3 |  |  |
| Staff member writes patient name on token | Porter | 0.2 |  |  |
| Patient registration | Receptionist | 4 |  |  |
| Assessing patient's financial status | Receptionist | 5 |  |  |
| Checking patient's vitals | Nurse | 2 |  |  |
| Initial medical evaluation | Medical doctor | 16 |  |  |
| Phlebotomy | Phlebotomist | 2 |  |  |
| Scheduling follow-up appointment | Receptionist | 5 |  |  |
| **Treatment initiation** |  |  |  |  |
| Visit confirmation | Receptionist | 3.5 | 2 | 5 |
| Triage – Vitals | Nurse | 6.5 | 5 | 8 |
| Medical consultation | Medical doctor | 17.5 | 15 | 20 |
| HBV vaccination | Nurse | 5 |  |  |
| Scheduling follow-up appointment | Receptionist | 5 | 3 | 7 |
| **Pharmacy** |  |  |  |  |
| Dispensing | Pharmacist | 3.5 | 2 | 5 |
| **Laboratory visit** |  |  |  |  |
| Visit confirmation | Receptionist | 2 |  |  |
| Phlebotomy | Phlebotomist | 3 |  |  |
| **Treatment follow-up** |  |  |  |  |
| Visit confirmation | Receptionist | 2 |  |  |
| Triage – Vitals | Nurse | 3 |  |  |
| Medical consultation | Medical doctor | 12.5 | 10 | 15 |
| HBV vaccination | Nurse | 5 |  |  |
| Scheduling follow-up appointment | Receptionist | 5 |  |  |
| **Treatment completion** |  |  |  |  |
| Visit confirmation | Receptionist | 2 |  |  |
| Triage – Vitals | Nurse | 3 |  |  |
| Medical consultation | Medical doctor | 5 |  |  |

Supplementary Table S6. Activities, resources and estimated unit costs.

Activities, resources and estimated unit costs in the Indus Hospital HCV treatment program. Costs are presented in 2019 US$.

| **Activity** | **Sub-activity** | **Ingredients** | **Type** | Unit cost |
| --- | --- | --- | --- | --- |
| Pre-treatment assessment | Patient receives token | Porter | Staff time | 0 |
|  | Staff member writes patient name on token | Porter | Staff time | 0 |
|  | Patient registration | Receptionist | Staff time | 0.06 |
|  | Assessing patient's financial status | Receptionist | Staff time | 0.08 |
|  | Checking patient's vitals | Nurse | Staff time | 0.07 |
|  | Initial medical evaluation | Medical doctor | Staff time | 1.44 |
|  | Phlebotomy | Phlebotomist | Staff time | 0.03 |
|  | Scheduling follow-up appointment | Receptionist | Staff time | 0.08 |
| Treatment initiation | Visit confirmation | Receptionist | Staff time | 0.06 |
|  | Triage – Vitals | Nurse | Staff time | 0.22 |
|  | Medical consultation | Medical doctor | Staff time | 1.57 |
|  | HBV vaccination | Nurse | Staff time | 0.17 |
|  | Scheduling follow-up appointment | Receptionist | Staff time | 0.08 |
| Medication pickup | Pharmacy dispensing | Pharmacist | Staff time | 0.1 |
| Laboratory tests | visit confirmation | Receptionist | Staff time | 0.03 |
|  | Phlebotomy | Phlebotomist | Staff time | 0.05 |
| Treatment follow-up | Visit confirmation | Receptionist | Staff time | 0.03 |
|  | Triage – Vitals | Nurse | Staff time | 0.1 |
|  | Medical consultation | Medical doctor | Staff time | 1.12 |
| End of treatment | HBV vaccination | Nurse | Staff time | 0.17 |
|  | Scheduling follow-up appointment | Receptionist | Staff time | 0.08 |
|  | Visit confirmation | Receptionist | Staff time | 0.03 |
|  | Triage – Vitals | Nurse | Staff time | 0.1 |
|  | Medical consultation | Medical doctor | Staff time | 0.45 |
| HCV clinic overheads | HCV clinic visit | HCV clinic | Space/Materials | 258.18 |

Supplementary Table S7. Estimated unit costs for clinic visits, laboratory tests and medicines.

Estimated unit costs for clinic visits, laboratory tests and medicines in the Indus Hospital HCV treatment program. Further breakdown of HCV clinic overheads are presented in Supplementary Table S8. Costs are presented in 2019 US$.

| **Resource** | **Unit cost (US$)** |
| --- | --- |
| **Clinic visits/consultations** | |
| Pre-treatment assessment | 1.76 |
| Treatment initiation | 2.09 |
| Medication pickup | 0.09 |
| Laboratory testing | 0.08 |
| Treatment follow-up | 1.50 |
| End of treatment | 1.50 |
| HCV clinic overheads | 258.18 |
| **Laboratory tests** | |
| CBC | 2.43 |
| LFTs | 2.43 |
| SGPT | 2.86 |
| SGOT | 4.15 |
| Creatinine | 2.22 |
| Serum albumin | 1.93 |
| Prothrombin time (PT) | 1.86 |
| Pregnancy test (urine) | 0.72 |
| Hepatitis C RNA | 16 |
| Hep B surface antigen | 2.93 |
| Hep B surface antibody | 5.29 |
| HIV | 3.07 |
| U/S Hepatobiliary | 7.87 |
| **DAA medicines (per tablet/day)** | |
| Sofosbuvir | 0.21 |
| Daclatasvir | 0.11 |
| Ribavarin | 0.05 |
| Peginterferon | 1.5 |
| Depo Medroxyprogesterone | 0.99 |

Supplementary Table S8. Breakdown for fixed costs for HCV clinic overheads.

Costs are presented in 2019 US$.

| **Cost Category** | **Allocated to HCV clinic (US$)** | **Allocated to HCV clinic (US$) per patient** | **% of clinic visit costs** |
| --- | --- | --- | --- |
| Support personnel costs | 58,423.04 | 45.39 | 17.12% |
| Coordination costs | 57,544.65 | 44.71 | 16.86% |
| Medical consultations | 214,182.10 | 166.42 | 62.75% |
| Utilities and bills | 1,957.37 | 1.52 | 0.57% |
| Non-medical equipment | 166.04 | 0.13 | 0.05% |
| **Total fixed cost allocated** | 332,252.1 | 258.18 | 97.35% |

Supplementary Table S9. Mean per-patient cost for clinic visits overall and by APRI score.

Costs are presented as mean (standard deviation) in 2019 US$.

| **Resource** | **APRI score** | | | | |
| --- | --- | --- | --- | --- | --- |
|  | **0-0.49** | **0.5-1.49** | **>=1.5** | **Missing** | **Total cohort** |
| Facility fixed | 258.18 (0.00) | 258.18 (0.00) | 258.18 (0.00) | 258.18 (0.00) | 258.18 (0.00) |
| Baseline assessment | 1.26 (0.48) | 1.27 (0.49) | 1.30 (0.54) | 1.15 (0.36) | 1.26 (0.48) |
| Adverse event follow-up | 0.00 (0.00) | 0.00 (0.00) | 0.01 (0.11) | 0.00 (0.00) | 0.00 (0.04) |
| Treatment initiation | 1.00 (0.00) | 1.00 (0.00) | 1.00 (0.00) | 1.00 (0.00) | 1.00 (0.00) |
| Treatment follow-up | 2.73 (1.04) | 3.03 (1.42) | 3.29 (1.84) | 2.61 (1.12) | 2.88 (1.29) |
| PCR follow-up | 0.27 (0.46) | 0.31 (0.46) | 0.24 (0.46) | 0.38 (0.49) | 0.29 (0.47) |
| Referred to specialist | 0.00 (0.00) | 0.00 (0.06) | 0.00 (0.00) | 0.01 (0.12) | 0.00 (0.05) |
| Treatment deferred | 0.08 (0.27) | 0.02 (0.15) | 0.01 (0.11) | 0.00 (0.00) | 0.04 (0.21) |
| End of treatment | 0.98 (0.15) | 0.98 (0.12) | 0.93 (0.25) | 0.92 (0.27) | 0.97 (0.17) |
| SVR12 result | 0.69 (0.50) | 0.76 (0.47) | 0.68 (0.50) | 0.70 (0.46) | 0.71 (0.49) |
| **TOTAL** |  |  |  |  |  |
| Abbreviations: APRI, Aspartate aminotransferase to Platelet Ratio Index; PCR, polymerase chain reaction; SVR, sustained virological response. | | | | | |

Supplementary Table S10. Resource use – HCV laboratory investigations.

| **Resource** | **APRI score** | | | | |
| --- | --- | --- | --- | --- | --- |
|  | **0-0.49** | **0.5-1.49** | **>=1.5** | **Missing** | **Total cohort** |
| Complete blood count | 2.16 (1.12) | 2.50 (1.17) | 3.00 (0.95) | 2.30 (1.16) | 2.37 (1.15) |
| Creatinine | 2.03 (0.86) | 2.11 (0.95) | 2.24 (0.98) | 2.00 (0.91) | 2.08 (0.91) |
| Hepatitis B surface antibody | 0.53 (0.50) | 0.54 (0.50) | 0.59 (0.49) | 0.47 (0.50) | 0.53 (0.50) |
| HCV genotyping | 0.21 (0.42) | 0.25 (0.46) | 0.24 (0.43) | 0.27 (0.48) | 0.23 (0.44) |
| Hepatitis B surface antigen | 0.82 (0.39) | 0.86 (0.35) | 0.80 (0.40) | 0.77 (0.43) | 0.83 (0.38) |
| HCV RNA | 1.87 (0.91) | 2.06 (0.92) | 1.97 (0.92) | 2.33 (1.07) | 1.99 (0.94) |
| HIV test | 0.62 (0.51) | 0.61 (0.50) | 0.50 (0.50) | 0.49 (0.53) | 0.59 (0.51) |
| Liver function tests | 0.94 (0.24) | 0.93 (0.25) | 0.91 (0.29) | 0.93 (0.25) | 0.93 (0.25) |
| Pregnancy test | 0.13 (0.39) | 0.06 (0.26) | 0.11 (0.31) | 0.04 (0.20) | 0.10 (0.33) |
| International normalized ratio | 1.12 (0.46) | 1.09 (0.36) | 1.16 (0.57) | 1.10 (0.53) | 1.11 (0.45) |
| Albumin | 1.10 (0.42) | 1.06 (0.37) | 1.01 (0.38) | 1.14 (0.90) | 1.08 (0.47) |
| Serum glutamic oxaloacetic transaminase | 1.27 (0.64) | 1.10 (0.37) | 1.04 (0.30) | 0.88 (0.55) | 1.16 (0.54) |
| Serum glutamic pyruvic transaminase | 1.08 (0.52) | 1.16 (0.66) | 1.37 (0.85) | 1.23 (0.77) | 1.15 (0.63) |

Abbreviations: APRI - Aspartate Aminotransferase to Platelet Ratio Index, HCV=Hepatitis C virus, HIV=Human immunodeficiency virus, RNA=Ribonucleic acid

**References**

International monetary fund. (2022, October 11, 2022). *Changes to the database - world economic outlook database*. Retrieved October from <https://www.imf.org/external/pubs/ft/weo/data/changes.htm>
